# IgA-Expressing Cells at the Human Ocular Surface and Reduced Tear IgA2 Abundance in Schirmer Strip Extracts from Patients with Dry Eye Disease and Primary Sjögren’s Syndrome

**DOI:** 10.3390/cells15211971

**Published:** 2026-10-29

**Authors:** Nils Schellekens, Jutta Horwath-Winter, Sophia Bittner, Wolfgang Schuh, Katharina Jüngert, Ingrid Zahn, Martin Schicht, Friedrich Paulsen

**Affiliations:** 1 Functional and Clinical Anatomy, Institute of Anatomy, Friedrich-Alexander-Universität Erlangen-Nürnberg (FAU), 91054 Erlangen, Germany; katharina.juengert@fau.de (K.J.); ingrid.zahn@fau.de (I.Z.); martin.schicht@fau.de (M.S.); 2 Department of Ophthalmology, Medical University of Graz, 8036 Graz, Austria; jutta.horwath@medunigraz.at; 3 Department of Translational Immunology, Department of Medicine 3—Rheumatology and Immunology, Nikolaus-Fiebiger-Center for Molecular Medicine, Friedrich-Alexander-Universität Erlangen-Nürnberg (FAU) and Uniklinikum Erlangen, 91054 Erlangen, Germany; sophia.bittner@fau.de (S.B.); wolfgang.schuh@uk-erlangen.de (W.S.); 4 Deutsches Zentrum Immuntherapie, Friedrich-Alexander-Universität Erlangen-Nürnberg (FAU) and Uniklinikum Erlangen, 91054 Erlangen, Germany

**Keywords:** ocular surface immunity, immunoglobulin A (IgA), IgA subclasses (IgA1/IgA2), dry eye disease (DED), primary Sjögren’s syndrome (pSS)

## Abstract

**Highlights:**

**What are the main findings?**

- IgA-expressing cells were identified in the human lacrimal gland and conjunctival stroma, with IgA1-expressing cells predominating over IgA2-expressing cells. No IgA-positive cells were detected in the examined corneal sections.
- Patients with dry eye disease (DED) and primary Sjögren’s syndrome (pSS) showed altered IgA subclass profiles in Schirmer strip extracts, with reduced normalized IgA2 abundance in both disease groups and reduced normalized total IgA abundance in pSS.

**What are the implications of the main findings?**

- The findings characterize the distribution of IgA-expressing cells in human ocular tissues and provide insights into the local IgA-associated mucosal immune environment.
- Altered IgA subclass profiles in Schirmer strip extracts suggest potential changes in ocular surface IgA homeostasis in DED and pSS, warranting further investigation of IgA production, epithelial transport, and regulation.

**Abstract:**

This study investigated the distribution of IgA-expressing cells in human ocular tissues and IgA subclass profiles in Schirmer strip extracts from patients with dry eye disease (DED) and primary Sjögren’s syndrome (pSS). Immunofluorescence staining was performed on paraffin-embedded lacrimal gland, conjunctival, and corneal tissues from body donors. Independently, Schirmer strip extracts from healthy controls (*n* = 18), patients with DED (*n *= 75), and patients with pSS (*n *= 10) were analyzed. Total protein concentrations were determined using a bicinchoninic acid assay, while total IgA, IgA1, and IgA2 were quantified by ELISA and normalized to total protein. CD38^+^ and CD38^−^ IgA-expressing cells were detected in the lacrimal gland and conjunctival stroma, with IgA1-expressing cells predominating over IgA2-expressing cells. No IgA-positive cells were detected in the examined corneal sections. Total protein concentrations were lower in pSS than in DED. Normalized IgA2 abundance was reduced in both DED and pSS compared with controls, while normalized total IgA was additionally reduced in pSS. Unnormalized extract concentrations showed broadly similar patterns. These independent findings demonstrate differences in recovered IgA subclass profiles, although differences in collection materials and patient characteristics preclude attributing these alterations exclusively to the underlying diseases.

## 1. Introduction

The ocular surface represents a highly integrated functional unit composed of the cornea, conjunctiva, eyelid glands, and lacrimal apparatus [1]. Together, these structures maintain tear film stability, provide optical quality, and protect the eye from environmental and microbial challenges. The tear film, which covers the ocular surface, is classically described as consisting of three functional layers: an outer lipid layer, an aqueous layer, and an inner mucin layer. The lipid layer, produced primarily by the meibomian glands, limits evaporation of the underlying aqueous phase [2]. The aqueous component is secreted mainly by the main and accessory lacrimal glands and contains oxygen, nutrients, antimicrobial peptides, and immunoglobulins that contribute to ocular surface defense [3]. The innermost mucin layer, generated predominantly by conjunctival goblet cells and epithelial cells, consists of membrane-bound and secreted mucins that promote tear film adhesion to the ocular surface and ensure uniform tear film distribution [4,5,6].

Disruption of tear film homeostasis can lead to dry eye disease (DED), a multifactorial disorder with an estimated prevalence ranging from 5% to 50% worldwide [7,8]. Clinically, patients experience symptoms such as ocular dryness, burning, irritation, and pain [8,9]. Pathophysiologically, DED is commonly categorized into evaporative dry eye (EDE), which is primarily associated with meibomian gland dysfunction and increased tear evaporation [6,10], and aqueous-deficient dry eye disease (ADDE), which arises from impaired lacrimal gland secretion. In many patients, both mechanisms coexist, leading to a mixed form of disease (MDE) [7,11].

In addition, DED frequently occurs in association with systemic autoimmune diseases, such as primary Sjögren’s syndrome (pSS), a chronic inflammatory disease characterized by lymphocytic infiltration and progressive dysfunction of the lacrimal and salivary glands [2,12].

The immunopathology of pSS and ocular surface inflammation has long been attributed primarily to T cell-mediated mechanisms. However, increasing evidence highlights a central role for B cells in disease initiation and progression [12,13]. Beyond their role as producers of antibodies and autoantibodies, B cells and their terminally differentiated counterparts, plasma cells, contribute to immune regulation through cytokine production and immunoglobulin secretion. This has raised growing interest in B cell-mediated immune responses at the ocular surface in health and disease [14].

Immunoglobulin A (IgA) is the predominant antibody class in mucosal secretions, including the tear film. Secretory IgA plays a critical role in ocular surface immunity by binding and neutralizing pathogens and preventing microbial adherence to epithelial surfaces [3,15]. In humans, IgA occurs in two subclasses, IgA1 and IgA2, which differ in their structural and glycosylation patterns and may exert distinct immunological functions; thus, IgA2 may have a pro-inflammatory effect on neutrophils, macrophages or dendritic cells [15,16,17]. IgA produced by plasmablasts and plasma cells in the lacrimal glands and conjunctival subepithelium is transported into the tear film via epithelial cells through the polymeric immunoglobulin receptor (pIgR), resulting in release of secretory IgA at the mucosal surface [18,19,20].

Previous studies have demonstrated local IgA production within human ocular tissues and have identified IgA-producing plasma cells in the lacrimal gland and other eye-associated lymphoid tissues. Moreover, the distribution of IgA subclasses in the human lacrimal gland has previously been investigated [21,22]. However, the distribution and relative abundance of IgA1- and IgA2-producing plasma cells across different ocular surface-associated tissues remain incompletely characterized. Furthermore, in contrast to other mucosal tissues such as the gastrointestinal tract in which plasma cells are localized in survival niches in the lamina propria, little is known about the relative abundance and localization of IgA plasma cells within the ocular surface tissues [19,20,23]. In the gastrointestinal tract, IgA plasma cells are generated in Peyer’s patches and mesenteric lymph nodes after sampling of intestinal antigens by M cells and dendritic cells, which activate naive B cells. With T cell help via CD40-CD40L and cytokines such as TGF-β, IL-21, and retinoic acid, B cells undergo class-switch recombination to IgA, differentiate into plasmablasts, and then migrate to the intestinal lamina propria, where they mature into IgA-secreting plasma cells [24]. Survival niches provide plasma cells with a microenvironment that supplies the cells with cytokines, survival factors, cell–cell contacts, and nutrients [25]. Whether comparable patterns of IgA-producing plasma cell localization occur across human ocular surface-associated tissues remains less well defined.

Alterations in IgA-mediated mucosal immunity may also be relevant to DED and pSS. Chronic ocular surface inflammation, tear film dysfunction, and, particularly in pSS, lacrimal gland pathology could be associated with changes in the amount or composition of IgA recovered from tears. However, comparatively little is known about IgA subclass profiles in tears from patients with DED and pSS. Importantly, alterations in tear IgA abundance may reflect several processes, including changes in IgA production, secretion, epithelial transport, degradation, or tear production, and therefore cannot by themselves identify the underlying mechanism.

Therefore, the present study aimed (1) to systematically map the distribution of IgA-, IgA1-, and IgA2-producing cells within human ocular surface tissues and (2) to determine whether the abundance of total IgA and its subclasses in Schirmer strip extracts differs between controls, patients with DED, and patients with pSS. By integrating tissue-based analyses with tear sample analyses, this study provides complementary information on IgA-mediated ocular surface immunity and its alterations in DED and pSS.

## 2. Materials and Methods

### 2.1. Collection and Fixation of Human Samples

Human lacrimal glands, eyelids, and corneas were obtained from body donors (Table 1) who had donated their bodies to the body donation program of the Institute of Anatomy, Friedrich-Alexander-Universität Erlangen-Nürnberg (FAU), Germany. All procedures involving human tissues were conducted in accordance with the principles of the Declaration of Helsinki. Written informed consent for the use of tissues for research purposes had been obtained from all body donors prior to death through the body donation program of the Institute of Anatomy, FAU, Germany. Donors with a history of recent trauma, ocular or nasal infection, or known diseases affecting lacrimal gland, eyelid, or corneal function were excluded. However, a prior history of dry eye disease (DED) in these donors cannot be completely excluded.

The tissues were carefully dissected within 24 h of death under anatomical guidance to avoid contamination with adjacent structures. Following excision, samples were fixed in paraformaldehyde (PFA) for 48 h, embedded in paraffin, and sectioned into 5 µm slices using a sliding microtome (Microm HM 430, Thermo Fisher Scientific, Waltham, MA, USA). Sections were mounted on microscope slides, and tissue morphology and structural integrity were confirmed by hematoxylin and eosin staining.

### 2.2. Immunofluorescence Staining

Paraffin sections were deparaffinized by incubation in xylene twice for 10 min, followed by rehydration through a descending ethanol series (100%, 100%, 100%, 96%, 96%, 80%, and 70%) for 3 min each. Sections were subsequently rinsed in deionized water for 5 min.

For antigen retrieval, slides were incubated in citrate buffer (pH 6.0) at 80 °C for 10 min and allowed to cool at room temperature for 3 h. Sections were then treated with trypsin (1 mg/mL; Sigma-Aldrich, St. Louis, MO, USA) diluted in Tris-buffered saline (TBS; 0.05 M Tris, 0.015 M NaCl, pH 7.3) for 10 min at 37 °C in a humidified chamber.

To reduce nonspecific antibody binding, sections were blocked for 1 h with skim milk powder (10 mg/mL; Merck, Darmstadt, Germany) in TBS containing Tween-20 (TBS-T; 1 µL/mL; Carl Roth, Karlsruhe, Germany). Slides were then incubated overnight at 4 °C in a humidified chamber with primary antibodies against CD38, IgA, IgA1, and IgA2. Primary antibodies were diluted in TBS-T supplemented with bovine serum albumin (BSA; 20 mg/mL; Carl Roth, Karlsruhe, Germany) and Triton X-100 (2 µL/mL; Carl Roth, Karlsruhe, Germany).

After incubation with the primary antibodies, sections were washed three times in TBS-T and subsequently incubated with Alexa Fluor 488- or Alexa Fluor 555-conjugated secondary antibodies for 1 h at room temperature. Slides were then washed three times in TBS-T and nuclei were counterstained with 4′,6-diamidino-2-phenylindole (DAPI; D9664, Merck, Darmstadt, Germany; 1:1000 dilution) for 3 min at room temperature.

Finally, sections were washed once with TBS-T, mounted with a fluorescent mounting medium (S3023; Agilent Technologies, Santa Clara, CA, USA), and covered with coverslips. Images were acquired using an inverted fluorescence phase-contrast microscope (Keyence BZ-X810, Keyence, Frankfurt am Main, Germany).

Negative controls, in which primary antibodies were omitted or replaced with isotype-matched control antibodies, were included in each staining run to assess nonspecific binding. Cells were classified according to their CD38 expression and staining for the respective IgA marker. CD38^+^IgA^+^, CD38^+^IgA1^+^, and CD38^+^IgA2^+^ cells were identified by visual assessment of merged fluorescence images, requiring both antibody-derived signals to be associated with the same DAPI-positive cell. Cells exhibiting staining for the respective IgA marker but no detectable CD38 staining were classified as CD38^−^IgA^+^, CD38^−^IgA1^+^, or CD38^−^IgA2^+^ cells. Classification was performed manually without predefined quantitative fluorescence intensity thresholds.

For each donor and tissue type, one tissue section was analyzed. Five randomly selected fields per section were imaged using a 40× objective, and double-positive cells were counted manually. The mean number of double-positive cells across the five fields was calculated and used as a single value per donor for subsequent analyses. Tissue samples were coded during image evaluation to minimize observer bias. As tissue types could be distinguished morphologically, complete blinding to tissue identity was not possible. Clinical disease status was unavailable for the tissue donors.

Corneal sections were systematically examined in their entirety for the presence of IgA-positive cells. Details of the primary and secondary antibodies are provided in Table 2 and Table 3.

### 2.3. Tear Fluid Samples

The collection and analysis of human tear fluid were approved by the local ethics committee of Friedrich-Alexander-Universität Erlangen-Nürnberg (FAU application number 25-273-B) and Medizinische Universität Graz (application number 32-015 ex 19/20) and conducted in accordance with the Declaration of Helsinki and good clinical practice. Written informed consent was obtained from all participants.

All participants completed the Ocular Surface Disease Index (OSDI) questionnaire and underwent a comprehensive ophthalmologic examination according to the guidelines of the Professional Association of Ophthalmologists in Germany (BVA). Control participants had no symptoms of DED, did not use artificial tears or ocular lubricants, had no autoimmune disorders or ocular diseases other than cataract or refractive errors, and had an OSDI score ≤ 12. Participants were classified as having DED if they had a documented diagnosis of DED for ≥6 months and an OSDI score ≥ 13. All DED patients were consecutively recruited during specialized dry eye consultation hours at the University Eye Clinic Graz.

DED patients were further categorized based on the Schirmer type 1 test and the presence of meibomian gland dysfunction (MGD) into aqueous-deficient dry eye (ADDE), evaporative dry eye (EDE), and mixed dry eye (MDE). Aqueous deficiency was defined as a Schirmer type 1 result < 10 mm/5 min in at least one eye. MGD was diagnosed based on slit-lamp examination, meibography, lipid layer analysis, and assessment of meibomian gland expressibility, as previously described [26]. MDE was defined by the presence of both aqueous deficiency and MGD. ADDE was defined as aqueous deficiency in the absence of MGD, whereas EDE was defined as MGD in the absence of aqueous deficiency. Ocular surface integrity was additionally assessed by tear film break-up time (BUT), National Eye Institute (NEI) corneal fluorescein staining, and Lissamine green staining of the conjunctiva.

The diagnosis of pSS was confirmed by the Department of Rheumatology and Immunology at the University Hospital in Graz according to the classification criteria described in [27]. Patients with allergic conjunctivitis, including atopic or vernal keratoconjunctivitis, rheumatoid arthritis, secondary Sjögren’s syndrome, facial nerve palsy, graft-versus-host disease, or other autoimmune diseases, including myasthenia gravis and vitiligo, were excluded to minimize potential confounding effects of concomitant inflammatory or autoimmune diseases on IgA and IgA subclass measurements.

Tear samples were collected from 103 participants (18 healthy, 75 DED, 10 pSS) using Schirmer strips without anesthetic (Schirmer type 1; Clement Clarke, Clement Clarke International Ltd., Mountain Ash, Wales, UK for DED and pSS patients; Optitech Mark Blu, Optitech Eyecare, Uttar Pradesh, India for controls). The resulting Schirmer strip extracts were used for subsequent protein and IgA analyses.

Following collection, Schirmer strips were immediately stored at −20 °C until extraction. Tear components were extracted from the Schirmer strips with 60 µL of 1× PBS by centrifugation at 13,000 rpm for 5 min at 4 °C, according to an adapted previously published protocol [28]. No protease inhibitors were added during extraction. Following extraction, samples were aliquoted and stored at −20 °C until analysis. Aliquots were thawed once and subsequently used for total protein and IgA analyses, thereby avoiding repeated freeze–thaw cycles.

For the present study, only samples obtained from the right eye of each participant were analyzed. DED classification, however, was based on the clinical criteria described above. Clinical and demographic characteristics of the study cohorts are summarized in Table 4.

Topical ocular and systemic immunomodulatory medications used by patients with DED and pSS were categorized according to their therapeutic class and are summarized in Supplementary Table S2. Topical treatments were classified as artificial tears and ocular lubricants, cyclosporine, corticosteroids, antibiotics, antiglaucoma medications, autologous serum eye drops, or albumin-containing eye drops. Systemic immunomodulatory treatments were recorded separately, while systemic medications without immunomodulatory activity and eyelid hygiene products were excluded from the analysis. Patients receiving multiple medications were assigned to each applicable category. Medication use is presented as the number of patients within each clinical group, including the DED subtypes. Healthy controls did not use any medication.

### 2.4. Total Protein Concentration in Schirmer Strip Extracts

Total protein concentrations in Schirmer strip extracts were measured using the Pierce BCA Protein Assay Kit (23227, Thermo Fisher Scientific, Waltham, MA, USA) following the manufacturer’s instructions. Standards (bovine serum albumin) and samples were loaded in duplicate (25 µL per well) on a 96-well F plate (82.1581, Sarstedt, Nümbrecht-Rommelsdorf, Germany). A total of 200 µL working reagent (reagent A:B, 50:1) was added per well, and the plate was shaken for 30 s and incubated at 37 °C for 30 min in the dark. After cooling to room temperature, absorbance was recorded at 562 nm using a CLARIOstar ELISA reader (BMG Labtech, Ortenberg, Germany). Total protein concentrations were determined from the BSA standard curve.

### 2.5. Quantification of Total IgA, IgA1 and IgA2 in Schirmer Strip Extracts

Total IgA, IgA1, and IgA2 in Schirmer strip extracts were quantified using in-house ELISAs. Briefly, 96-well F plates (82.1581, Sarstedt, Nümbrecht-Rommelsdorf, Germany) were coated overnight at 4 °C with F(ab’)_2_ anti-IgA antibody diluted in carbonate-bicarbonate coating buffer containing 15 mM Na_2_CO_3_ and 35 mM NaHCO_3_. Plates were washed three times with washing buffer containing 10% PBS and 0.05% Tween-20 and blocked with PBS containing 2% FCS for 1 h at room temperature.

Human IgA subclass serum (5691554, antibodies-online, Limerick, PA, USA), with manufacturer-assigned concentrations of total IgA, IgA1, and IgA2, was used as reference material for the respective standard curves. Standards and samples were applied in duplicate (100 µL per well), with PBS containing 2% FCS serving as blank, and incubated for 1.5 h at room temperature. After three washing steps, bound IgA was detected using HRP-conjugated anti-human IgA, IgA1, or IgA2 antibodies for 1 h at room temperature. Antibody details and working concentrations are provided in Table 5. Following three additional washing steps, 100 µL of TMB substrate (555214, BD, Franklin Lakes, NJ; USA) was added to each well. The reaction was stopped by addition of 100 µL of 0.5 M sulfuric acid, and absorbance was measured at 450 nm using a CLARIOstar ELISA reader (BMG Labtech, Ortenberg, Germany).

Concentrations were calculated from the respective standard curves using four-parameter logistic (4PL) regression. During initial assay establishment, additional dilutions of the reference serum were analyzed as test samples using the respective ELISAs. The measured concentrations were in approximate agreement with the expected concentrations calculated from the manufacturer-assigned values. As the same reference serum was used for calibration and testing, this procedure served as an initial assay performance check rather than an independent validation of analytical accuracy. The subclass specificity of the IgA1 and IgA2 detection antibodies was based on the manufacturers’ documentation.

For all 103 samples included in the study, concentrations of total IgA, IgA1, and IgA2 were within the respective standard curve ranges; therefore, no extrapolation beyond the standard curves was required. For the primary analyses, IgA concentrations were normalized to the total protein concentration determined in the corresponding Schirmer strip extract and expressed as µg of the respective IgA analyte per mg total protein. In addition, unnormalized IgA concentrations in Schirmer strip extracts were analyzed and are presented in Supplementary Figure S8. These values represent concentrations in the extracts rather than absolute concentrations in native tear fluid.

### 2.6. Statistics

Data are presented as mean ± standard error of the mean (SEM). Statistical analyses were performed using GraphPad Prism 11 (version 11.0.0; GraphPad Software, San Di-ego, CA, USA). Data distribution was assessed using the Kolmogorov–Smirnov normality test. Outliers were identified using the ROUT method with a Q value of 1% and excluded from the respective analyses. For comparisons between independent groups, normally distributed data were analyzed using one-way analysis of variance (ANOVA) followed by Tukey’s multiple comparisons test. For data that did not meet the assumptions of parametric testing, the Kruskal–Wallis test followed by Dunn’s multiple comparisons test was applied. Multiple comparisons within each analysis were adjusted using the respective post hoc procedure. To assess the robustness of the observed differences in normalized IgA2 abundance, an additional sensitivity analysis was performed including all observations previously excluded by the ROUT method (Q = 1%). Data distribution was assessed using the Kolmogorov–Smirnov test, and group differences were evaluated using the Kruskal–Wallis test followed by Dunn’s multiple comparisons test. The results of the sensitivity analysis are presented in Supplementary Table S3. To explore potential associations of demographic factors with total protein and IgA measurements, additional analyses stratified by sex and age (≤50 years and >50 years) were performed. These analyses included total protein as well as total IgA, IgA1, and IgA2 measurements normalized to total protein. Given small and unequal subgroup sizes, particularly in the pSS cohort (*n *= 10), as well as differences in the demographic composition of the study cohorts, these analyses were considered exploratory and hypothesis-generating rather than confirmatory. Associations between clinical parameters (OSDI, Schirmer test, tear film break-up time [BUT], National Eye Institute [NEI] score, and Lissamine green staining score) and normalized total IgA, IgA1, and IgA2 measurements were assessed using Spearman’s rank correlation coefficient. To account for multiple hypothesis testing across the exploratory correlation analyses, *p*-values were adjusted using the Benjamini–Hochberg procedure to control the false discovery rate (FDR) at 5%. Separate test families were defined for each combination of clinical parameter (OSDI, Schirmer test, BUT, NEI score, and Lissamine green staining score) and IgA parameter (total IgA, IgA1, IgA2, and the IgA1/IgA2 ratio), resulting in a total of 20 test families. Each test family comprised the correlations across the investigated study groups and age strata for the respective combination of clinical and IgA parameters. A total of 196 correlation tests were included across all 20 test families. Unadjusted and FDR-adjusted *p*-values for correlations reaching nominal significance (unadjusted *p* < 0.05) are reported in Supplementary Table S1. Statistical significance was defined as *p* ≤ 0.05. Significance levels for the group comparisons are indicated as follows: * *p* < 0.05, ** *p* < 0.01, *** *p* < 0.001, and **** *p* < 0.0001.

## 3. Results

### 3.1. Occurrence and Spatial Distribution of IgA and IgA Subclass-Producing Positive Cells in Tissues of the Ocular Surface

**Figure 1:**
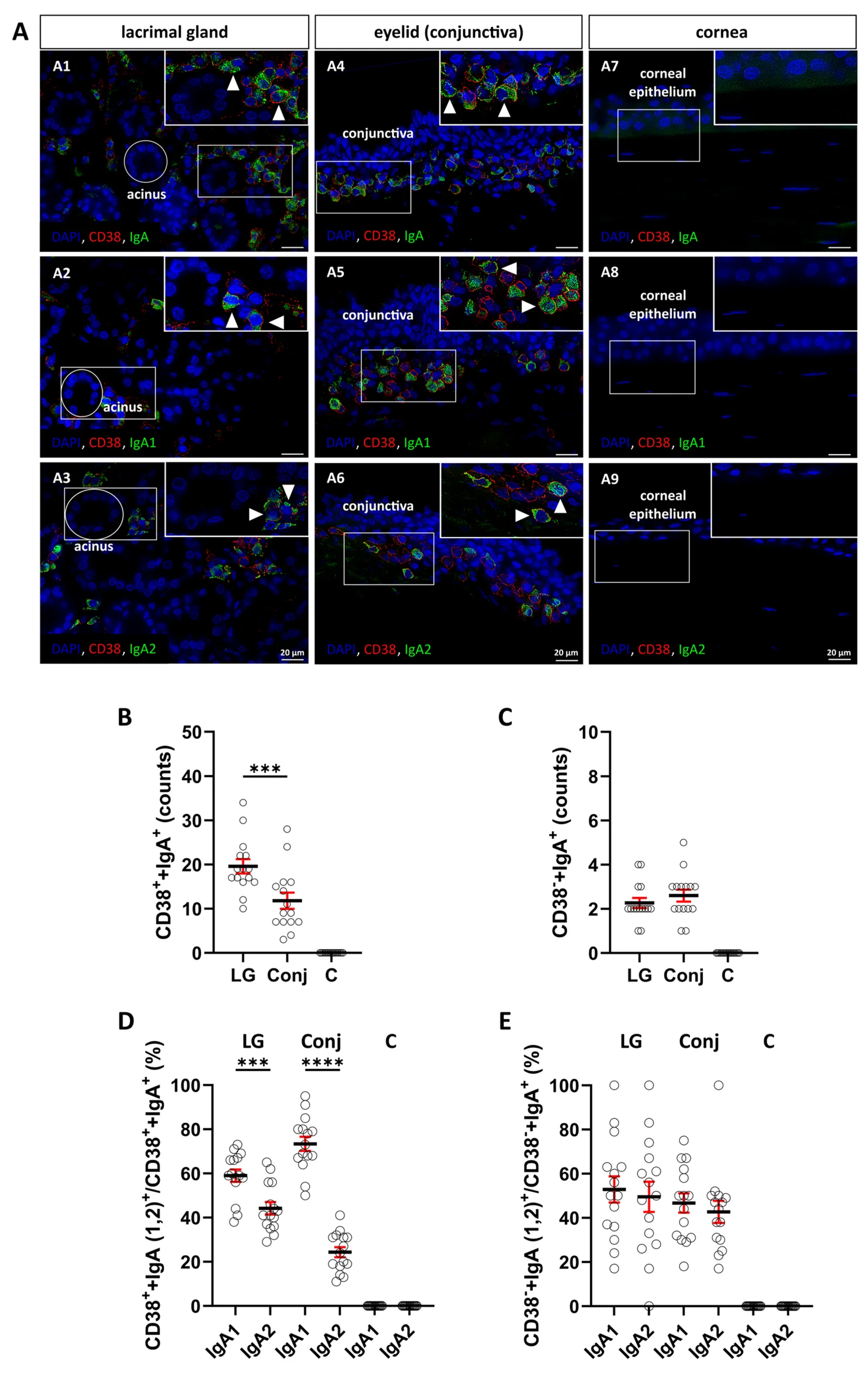
Immunofluorescence detection and quantification of CD38^+^ and CD38^−^ IgA-expressing cells in the human lacrimal gland, conjunctiva and cornea. (**A**) Representative immunofluorescence images showing CD38 and IgA staining in the lacrimal gland (**A1**–**A3**), eyelid with conjunctiva (**A4**–**A6**), and cornea with corneal epithelium (**A7**–**A9**). Images were obtained from tissue sections of different human body donors (Table 1). Insets show higher-magnification views. Arrowheads indicate representative IgA-positive cells. Scale bar: 20 µm. (**B**) Quantification of CD38^+^IgA^+^ cells in the lacrimal gland (LG), eyelid with conjunctiva (Conj), and cornea (**C**), presented as cell counts. (**C**) Quantification of CD38^−^IgA^+^ cells in the corresponding tissues, presented as cell counts. (**D**) Quantification of CD38^+^IgA1^+^ and CD38^+^IgA2^+^ cells in the LG, Conj, and C, expressed as percentages of the corresponding IgA-positive cell population. (**E**) Quantification of CD38^−^IgA1^+^ and CD38^−^IgA2^+^ cells in the corresponding tissues, expressed as percentages of the corresponding IgA-positive cell population. For each donor and tissue type, one tissue section was analyzed. Five randomly selected microscopic fields per section were imaged using a 40× objective, and the mean value across the five fields was calculated. Each data point represents one donor. Corneal sections were additionally examined in their entirety for the presence of IgA-positive cells. *** *p* < 0.001, **** *p* < 0.0001.

Immunofluorescence staining was performed on PFA-fixed, paraffin-embedded tissues from human body donors (Table 1) to investigate the occurrence and spatial distribution of CD38^+^IgA^+^, CD38^+^IgA1^+^, and CD38^+^IgA2^+^ cells in the lacrimal gland, conjunctival region, and cornea. Double-positive cells were identified based on the colocalization of CD38 and the respective IgA marker within the same DAPI-positive cell. CD38^+^IgA^+^ cells, including cells expressing IgA1 or IgA2, were observed in the connective tissue of the lacrimal gland, particularly in close proximity to the acini (Figure 1(A1–A3)), and in the eyelid connective tissue beneath the conjunctiva (Figure 1(A4–A6)). In contrast, no IgA-positive cells were detected in the examined corneal sections (Figure 1(A7–A9)). Quantification based on five randomly selected microscopic fields per tissue section revealed significantly higher numbers of CD38^+^IgA^+^ cells in the lacrimal gland than in the eyelid connective tissue (Figure 1A). Furthermore, CD38^+^IgA1^+^ cells significantly outnumbered CD38^+^IgA2^+^ cells in both the lacrimal gland (IgA1/IgA2 ratio: 1.2) and the eyelid connective tissue (IgA1/IgA2 ratio: 3.0; Figure 1D). CD38^+^IgA2^+^ cells were detected only infrequently in the eyelid connective tissue beneath the conjunctiva (Figure 1D).

### 3.2. Reduced Total Protein Concentrations and IgA Abundance in Schirmer Strip Extracts from Patients with Primary Sjögren’s Syndrome

**Figure 2:**
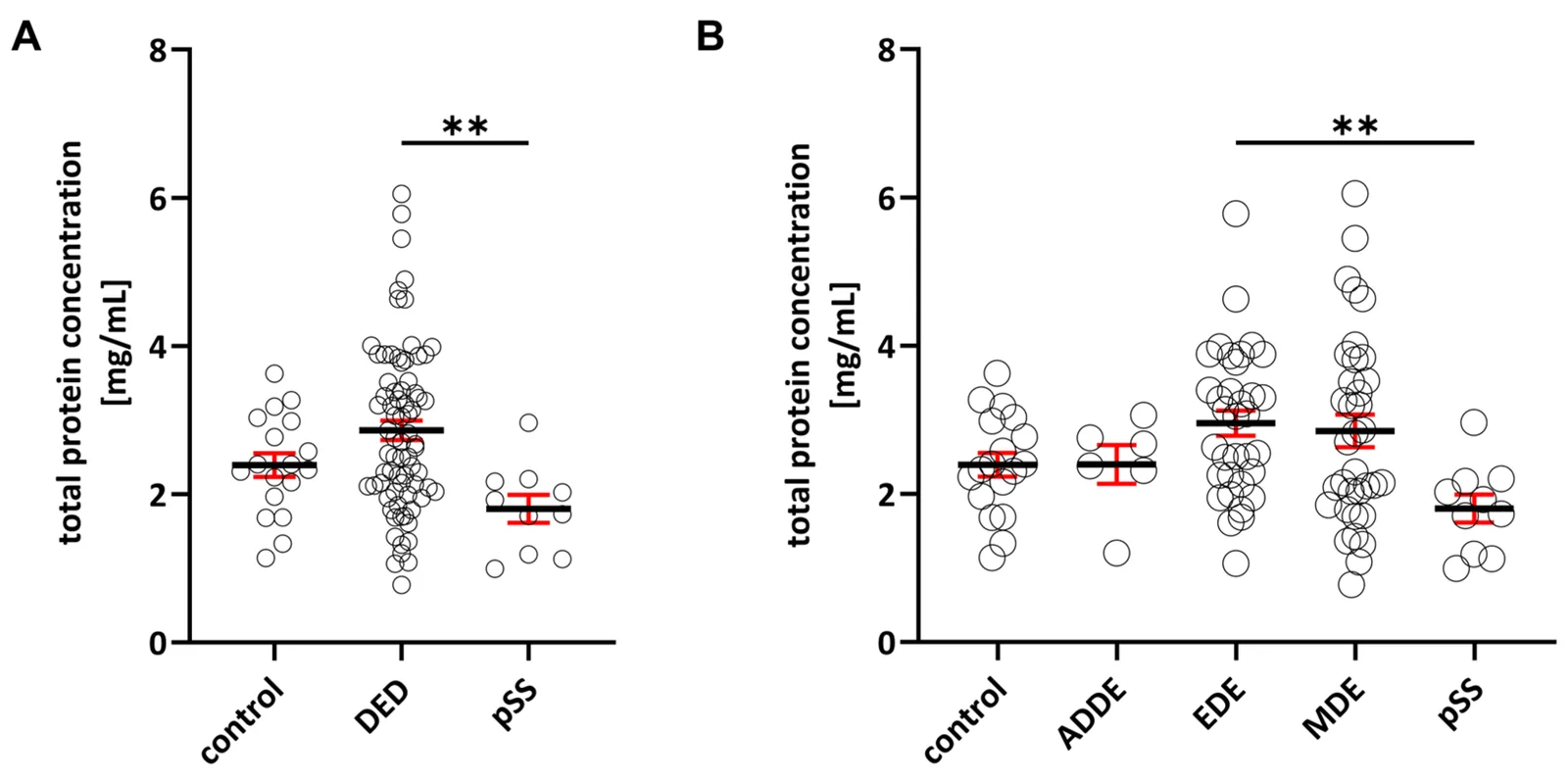
Total protein concentrations in Schirmer strip extracts from controls and patients with DED and pSS. (**A**) Comparison of total protein concentrations between controls and patients with DED or pSS. Total protein concentrations were significantly higher in patients with DED than in patients with pSS. Sample sizes after outlier exclusion: controls (*n *= 18; 0 outliers excluded), DED (*n *= 75; 0 outliers excluded), and pSS (*n *= 10; 0 outliers excluded). (**B**) Exploratory subgroup analysis showing significantly higher total protein concentrations in patients with EDE compared with patients with pSS. Sample sizes after outlier exclusion: controls (*n *= 18; 0 outliers excluded), ADDE (*n *= 6; 0 outliers excluded), EDE (*n *= 34; 0 outliers excluded), MDE (*n *= 35; 0 outliers excluded), and pSS (*n *= 10; 0 outliers excluded). Each data point represents the total protein concentration measured in the Schirmer strip extract of an individual participant. ** *p* < 0.01.

Schirmer strip samples were collected from controls and patients with DED and pSS. Total protein concentrations in Schirmer strip extracts were determined using a BCA assay. Patients with pSS showed significantly lower protein concentrations than patients with DED, whereas no significant difference was observed between DED patients and controls (Figure 2A). Exploratory analyses of DED subtypes revealed significantly higher protein concentrations in patients with EDE than in patients with pSS (Figure 2B). Exploratory sex-stratified analyses showed significantly higher protein concentrations in female patients with DED than in female patients with pSS, whereas no significant differences were detected following further stratification by DED subtype (Figure S1). Exploratory age-stratified analyses revealed no significant differences in total protein concentrations (Figure S2).

**Figure 3:**
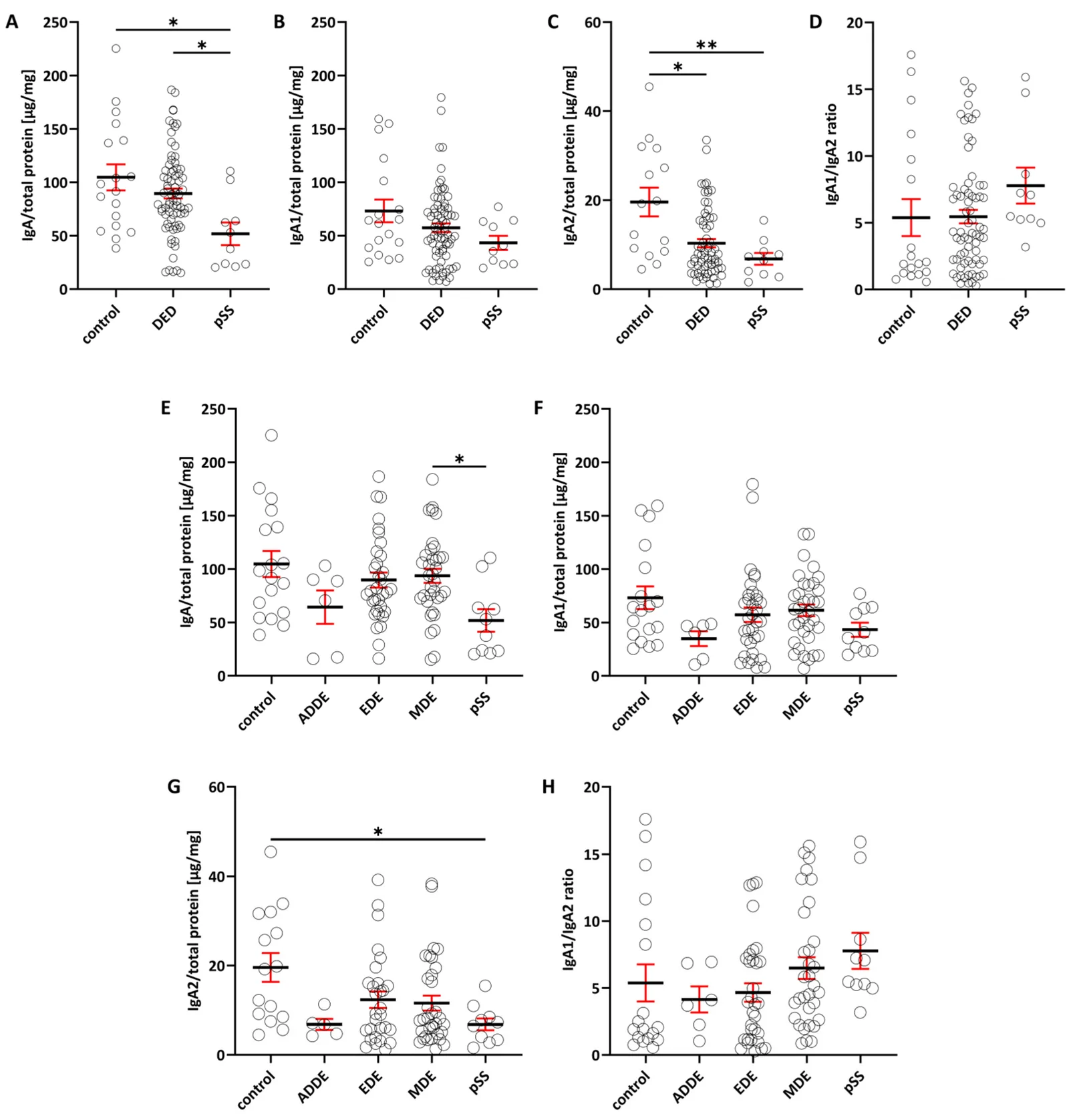
ELISA analysis of total IgA, IgA1 and IgA2 in Schirmer strip extracts normalized to total protein content. (**A**–**D**) Comparison of normalized total IgA, IgA1, and IgA2 abundance and the IgA1/IgA2 ratio between controls and patients with DED or pSS. Normalized total IgA abundance was significantly lower in patients with pSS than in controls and patients with DED, whereas normalized IgA2 abundance was significantly lower in patients with DED and pSS than in controls. Sample sizes after outlier exclusion: (**A**) controls (*n *= 18; 0 outliers excluded), DED (*n *= 74; 1 outlier excluded), and pSS (*n *= 10; 0 outliers excluded); (**B**) controls (*n *= 18; 0 outliers excluded), DED (*n *= 75; 0 outliers excluded), and pSS (*n *= 10; 0 outliers excluded); (**C**) controls (*n *= 15; 3 outliers excluded), DED (*n *= 65; 10 outliers excluded), and pSS (*n *= 10; 0 outliers excluded); (**D**) controls (*n *= 18; 0 outliers excluded), DED (*n *= 70; 5 outliers excluded), and pSS (*n *= 10; 0 outliers excluded). (**E**–**H**) Exploratory subgroup analysis of DED patients revealed significantly lower normalized IgA2 abundance in patients with pSS compared with controls and significantly lower normalized total IgA abundance in patients with pSS compared with patients with MDE. Sample sizes after outlier exclusion: (**E**) controls (*n *= 18; 0 outliers excluded), ADDE (*n *= 6; 0 outliers excluded), EDE (*n *= 33; 1 outlier excluded), MDE (*n *= 35; 0 outliers excluded), and pSS (*n *= 10; 0 outliers excluded); (**F**) controls (*n *= 18; 0 outliers excluded), ADDE (*n *= 6; 0 outliers excluded), EDE (*n *= 34; 0 outliers excluded), MDE (*n *= 35; 0 outliers excluded), and pSS (*n *= 10; 0 outliers excluded); (**G**) controls (*n *= 15; 3 outliers excluded), ADDE (*n *= 5; 1 outlier excluded), EDE (*n *= 29; 5 outliers excluded), MDE (*n *= 34; 1 outlier excluded), and pSS (*n *= 10; 0 outliers excluded); (**H**) controls (*n *= 18; 0 outliers excluded), ADDE (*n *= 6; 0 outliers excluded), EDE (*n *= 32; 2 outliers excluded), MDE (*n *= 32; 3 outliers excluded), and pSS (*n *= 10; 0 outliers excluded). Each data point represents one individual participant. * *p* < 0.05, ** *p* < 0.01.

Total IgA, IgA1, and IgA2 were quantified by ELISA and normalized to total protein content. Normalized total IgA abundance was significantly lower in patients with pSS than in controls and patients with DED, whereas normalized IgA2 abundance was significantly lower in both DED and pSS compared with controls. No significant differences were observed for normalized IgA1 abundance or the IgA1/IgA2 ratio (Figure 3A–D). Exploratory analyses of DED subtypes showed significantly lower normalized total IgA abundance in pSS than in MDE and lower normalized IgA2 abundance in pSS than in controls, without additional significant differences (Figure 3E–H). To assess the robustness of the normalized IgA2 findings, a sensitivity analysis was performed including all observations previously excluded as outliers. The differences between controls and DED (*p* = 0.0245) and between controls and pSS (*p* = 0.0040) remained statistically significant, whereas the difference between DED and pSS remained nonsignificant (*p* = 0.2683). Thus, the statistical significance of the main IgA2 findings was maintained when previously excluded observations were included (Supplementary Table S3).

To complement the normalized measurements, unnormalized ELISA concentrations in Schirmer strip extracts were also analyzed (Figure S8). Total IgA and IgA2 concentrations were significantly lower in pSS than in controls, while total IgA, IgA1, and IgA2 concentrations were lower in pSS than in DED. In addition, IgA2 concentrations were significantly lower in DED than in controls (Figure S8A–C). Exploratory DED subtype analyses showed lower total IgA concentrations in pSS than in controls, EDE, and MDE, as well as lower IgA2 concentrations in pSS than in controls and EDE (Figure S8D–F). Although the overall patterns were broadly comparable, the significance of individual comparisons differed between normalized and unnormalized measurements.

**Figure 4:**
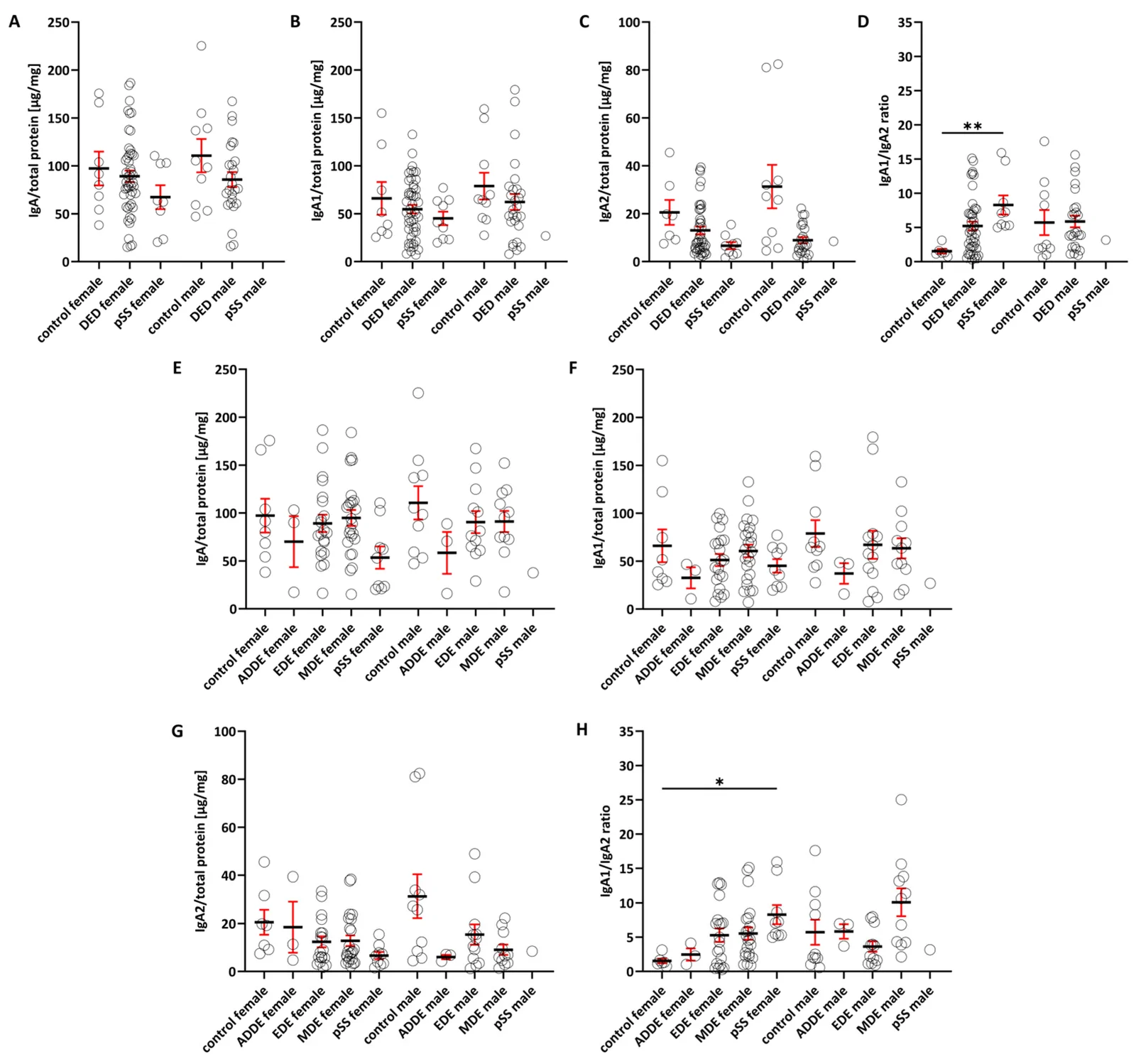
ELISA analysis of total IgA, IgA1, and IgA2 in Schirmer strip extracts normalized to total protein content, stratified by sex. (**A**–**D**) Exploratory sex-stratified analysis comparing normalized total IgA, IgA1, and IgA2 abundance and the IgA1/IgA2 ratio between controls and patients with DED or pSS. The IgA1/IgA2 ratio was significantly higher in female patients with pSS than in female controls. Sample sizes after outlier exclusion: (**A**) female controls (*n *= 8; 0 outliers excluded), female DED (*n *= 49; 0 outliers excluded), female pSS (*n *= 8; 1 outlier excluded), male controls (*n *= 10; 0 outliers excluded), male DED (*n *= 25; 1 outlier excluded), and male pSS (*n *= 0; 1 outlier excluded); (**B**) female controls (*n *= 8; 0 outliers excluded), female DED (*n *= 48; 0 outliers excluded), female pSS (*n *= 9; 0 outliers excluded), male controls (*n *= 10; 0 outliers excluded), male DED (*n *= 27; 0 outliers excluded), and male pSS (*n *= 1; 0 outliers excluded); (**C**) female controls (*n *= 7; 1 outlier excluded), female DED (*n *= 44; 4 outliers excluded), female pSS (*n *= 9; 0 outliers excluded), male controls (*n *= 10; 0 outliers excluded), male DED (*n *= 24; 3 outliers excluded), and male pSS (*n *= 1; 0 outliers excluded); (**D**) female controls (*n *= 6; 2 outliers excluded), female DED (*n *= 45; 4 outliers excluded), female pSS (*n *= 9; 0 outliers excluded), male controls (*n *= 10; 0 outliers excluded), male DED (*n *= 25; 2 outliers excluded), and male pSS (*n *= 1; 0 outliers excluded). (**E**–**H**) Exploratory sex-stratified subgroup analysis of DED patients showed significantly higher IgA1/IgA2 ratio in female patients with pSS than in female controls. Sample sizes after outlier exclusion: (**E**) female controls (*n *= 8; 0 outliers excluded), female ADDE (*n *= 3; 0 outliers excluded), female EDE (*n *= 21; 0 outliers excluded), female MDE (*n *= 24; 0 outliers excluded), female pSS (*n *= 9; 0 outliers excluded), male controls (*n *= 10; 0 outliers excluded), male ADDE (*n *= 3; 0 outliers excluded), male EDE (*n *= 12; 1 outlier excluded), male MDE (*n *= 11; 0 outliers excluded), and male pSS (*n *= 1; 0 outliers excluded); (**F**) female controls (*n *= 8; 0 outliers excluded), female ADDE (*n *= 3; 0 outliers excluded), female EDE (*n *= 21; 0 outliers excluded), female MDE (*n *= 24; 0 outliers excluded), female pSS (*n *= 9; 0 outliers excluded), male controls (*n *= 10; 0 outliers excluded), male ADDE (*n *= 3; 0 outliers excluded), male EDE (*n *= 13; 0 outliers excluded), male MDE (*n *= 11; 0 outliers excluded), and male pSS (*n *= 1; 0 outliers excluded); (**G**) female controls (*n *= 7; 1 outlier excluded), female ADDE (*n *= 3; 0 outliers excluded), female EDE (*n *= 18; 3 outliers excluded), female MDE (*n *= 23; 1 outlier excluded), female pSS (*n *= 9; 0 outliers excluded), male controls (*n *= 10; 0 outliers excluded), male ADDE (*n *= 3; 0 outliers excluded), male EDE (*n *= 12; 1 outlier excluded), male MDE (*n *= 11; 0 outliers excluded), and male pSS (*n *= 1; 0 outliers excluded); (**H**) female controls (*n *= 6; 2 outliers excluded), female ADDE (*n *= 3; 0 outliers excluded), female EDE (*n *= 20; 1 outlier excluded), female MDE (*n *= 22; 2 outliers excluded), female pSS (*n *= 9; 0 outliers excluded), male controls (*n *= 10; 0 outliers excluded), male ADDE (*n *= 3; 0 outliers excluded), male EDE (*n *= 12; 1 outlier excluded), male MDE (*n *= 11; 0 outliers excluded), and male pSS (*n *= 1; 0 outliers excluded). Each data point represents an individual participant. All sex-stratified analyses were exploratory and should be interpreted cautiously because of the small and unequal subgroup sizes, particularly in the pSS cohort. * *p* < 0.05, ** *p* < 0.01.

Exploratory sex-stratified analyses of normalized IgA measurements revealed a significantly higher IgA1/IgA2 ratio in female patients with pSS than in female controls, both in the overall DED comparison and following subdivision into DED subtypes (Figure 4A–H). No other significant differences were detected. Given the small and unequal subgroup sizes, these observations should be considered hypothesis-generating rather than evidence of sex-specific alterations in IgA subclass profiles.

**Figure 5:**
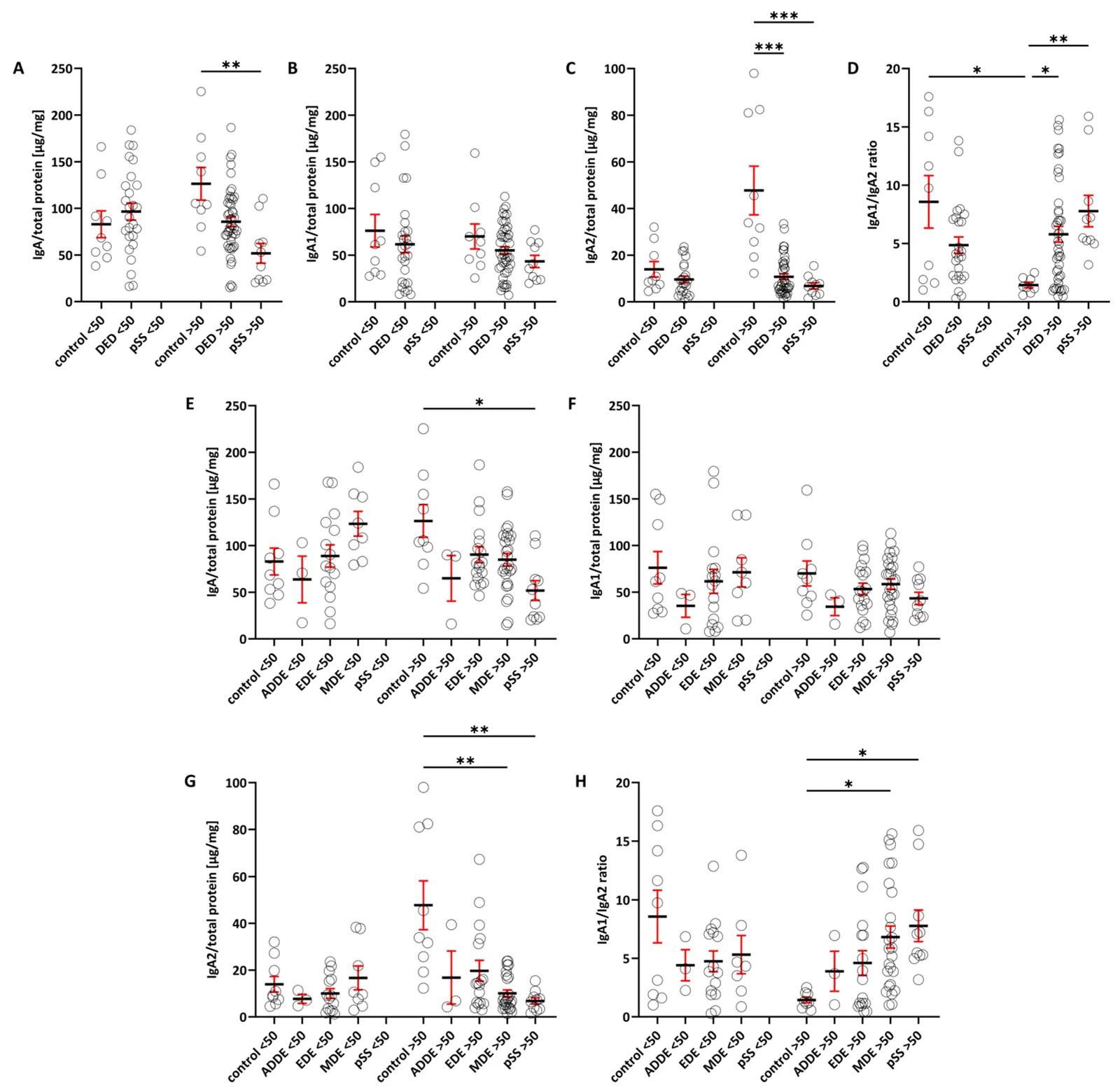
ELISA analysis of total IgA, IgA1, and IgA2 in Schirmer strip extracts normalized to total protein content, stratified by age. (**A**–**D**) Exploratory age-stratified analysis comparing normalized total IgA, IgA1, and IgA2 abundance and the IgA1/IgA2 ratio between controls and patients with DED or pSS. Among participants aged >50 years, normalized total IgA and IgA2 abundance were significantly lower in patients with pSS than in age-matched controls. Normalized IgA2 abundance was also significantly lower in patients with DED aged >50 years than in age-matched controls. The IgA1/IgA2 ratio was significantly higher in patients with DED and pSS aged >50 years than in age-matched controls. Additionally, controls aged >50 years exhibited a significantly lower IgA1/IgA2 ratio than controls aged ≤50 years. Sample sizes after outlier exclusion: (**A**) controls ≤ 50 years (*n *= 9; 0 outliers excluded), controls > 50 years (*n *= 9; 0 outliers excluded), DED ≤ 50 years (*n *= 26; 1 outlier excluded), DED > 50 years (*n *= 48; 0 outliers excluded), pSS ≤ 50 years (*n *= 0; 0 outliers excluded), and pSS > 50 years (*n *= 10; 0 outliers excluded); (**B**) controls ≤50 years (*n *= 9; 0 outliers excluded), controls > 50 years (*n *= 9; 0 outliers excluded), DED ≤ 50 years (*n *= 27; 0 outliers excluded), DED > 50 years (*n *= 48; 0 outliers excluded), pSS ≤ 50 years (*n *= 0; 0 outliers excluded), and pSS > 50 years (*n *= 10; 0 outliers excluded); (**C**) controls ≤ 50 years (*n *= 9; 0 outliers excluded), controls > 50 years (*n *= 9; 0 outliers excluded), DED ≤ 50 years (*n *= 23; 4 outliers excluded), DED > 50 years (*n *= 42; 6 outliers excluded), pSS ≤ 50 years (*n *= 0; 0 outliers excluded), and pSS > 50 years (*n *= 10; 0 outliers excluded); (**D**) controls ≤ 50 years (*n *= 9; 0 outliers excluded), controls > 50 years (*n *= 8; 1 outlier excluded), DED ≤ 50 years (*n *= 25; 2 outliers excluded), DED > 50 years (*n *= 45; 3 outliers excluded), pSS ≤ 50 years (*n *= 0; 0 outliers excluded), and pSS > 50 years (*n *= 10; 0 outliers excluded). (**E**–**H**) Exploratory age-stratified subgroup analysis of DED patients showed among participants aged >50 years, significantly lower normalized total IgA and IgA2 abundance in patients with pSS than in age-matched controls. Normalized IgA2 abundance was also significantly lower in patients with MDE aged >50 years than in age-matched controls. The IgA1/IgA2 ratio was significantly higher in patients with MDE and pSS aged >50 years than in age-matched controls. Sample sizes after outlier exclusion: (**E**) controls ≤ 50 years (*n *= 9; 0 outliers excluded), controls > 50 years (*n *= 9; 0 outliers excluded), ADDE ≤ 50 years (*n *= 3; 0 outliers excluded), ADDE > 50 years (*n *= 3; 0 outliers excluded), EDE ≤ 50 years (*n *= 15; 1 outlier excluded), EDE > 50 years (*n *= 18; 0 outliers excluded), MDE ≤ 50 years (*n *= 8; 0 outliers excluded), MDE > 50 years (*n *= 27; 0 outliers excluded), pSS ≤ 50 years (*n *= 0; 0 outliers excluded), and pSS > 50 years (*n *= 10; 0 outliers excluded); (**F**) controls ≤ 50 years (*n *= 9; 0 outliers excluded), controls > 50 years (*n *= 9; 0 outliers excluded), ADDE ≤ 50 years (*n *= 3; 0 outliers excluded), ADDE > 50 years (*n *= 3; 0 outliers excluded), EDE ≤ 50 years (*n *= 16; 0 outliers excluded), EDE > 50 years (*n *= 18; 0 outliers excluded), MDE ≤ 50 years (*n *= 8; 0 outliers excluded), MDE > 50 years (*n *= 27; 0 outliers excluded), pSS ≤ 50 years (*n *= 0; 0 outliers excluded), and pSS > 50 years (*n *= 10; 0 outliers excluded); (**G**) controls ≤ 50 years (*n *= 9; 0 outliers excluded), controls > 50 years (*n *= 9; 0 outliers excluded), ADDE ≤ 50 years (*n *= 3; 0 outliers excluded), ADDE > 50 years (*n *= 3; 0 outliers excluded), EDE ≤ 50 years (*n *= 14; 2 outliers excluded), EDE > 50 years (*n *= 17; 1 outlier excluded), MDE ≤ 50 years (*n *= 8; 0 outliers excluded), MDE > 50 years (*n *= 26; 1 outlier excluded), pSS ≤ 50 years (*n *= 0; 0 outliers excluded), and pSS > 50 years (*n *= 10; 0 outliers excluded); (**H**) controls ≤ 50 years (*n *= 9; 0 outliers excluded), controls > 50 years (*n *= 8; 1 outlier excluded), ADDE ≤ 50 years (*n *= 3; 0 outliers excluded), ADDE > 50 years (*n *= 3; 0 outliers excluded), EDE ≤ 50 years (*n *= 15; 1 outlier excluded), EDE > 50 years (*n *= 17; 1 outlier excluded), MDE ≤ 50 years (*n *= 7; 1 outlier excluded), MDE > 50 years (*n *= 25; 2 outliers excluded), pSS ≤ 50 years (*n *= 0; 0 outliers excluded), and pSS > 50 years (*n *= 10; 0 outliers excluded). Each data point represents an individual participant. All age-stratified analyses were exploratory. No patients with pSS were aged ≤50 years, preventing comparisons between age groups within the pSS cohort. Findings from the smaller subgroups should be interpreted cautiously. * *p* < 0.05, ** *p* < 0.01, *** *p* < 0.001.

Exploratory age-stratified analyses showed significantly lower normalized total IgA abundance in patients with pSS aged >50 years and lower normalized IgA2 abundance in patients with DED or pSS aged >50 years compared with age-matched controls. The IgA1/IgA2 ratio was significantly higher in both disease groups aged >50 years than in controls of the same age, whereas controls aged > 50 years exhibited a lower ratio than controls aged ≤50 years (Figure 5A–D). Further exploratory stratification by DED subtype showed lower normalized total IgA and IgA2 abundance in pSS and lower normalized IgA2 abundance in MDE among participants aged >50 years compared with age-matched controls. The IgA1/IgA2 ratio was also higher in MDE and pSS patients aged >50 years than in age-matched controls (Figure 5E–H). As no patients with pSS were aged ≤50 years, these comparisons do not allow conclusions regarding age-related changes within the pSS group.

Exploratory Spearman correlation analyses identified several nominally significant associations between normalized IgA parameters and clinical measures, including OSDI, Schirmer test values, tear film break-up time (BUT), National Eye Institute (NEI) scores, and Lissamine green staining scores (Figures S3–S7). After Benjamini–Hochberg correction for multiple testing, performed separately for each combination of clinical and IgA parameters, only two correlations remained significant (FDR = 5%). Specifically, the IgA1/IgA2 ratio was negatively correlated with both OSDI scores and Schirmer test values in controls aged ≤50 years (Figures S3 and S4; Table S1). All other nominally significant correlations did not remain significant after FDR correction.

Overall, patients with pSS showed lower total protein concentrations in Schirmer strip extracts than patients with DED. Normalized total IgA abundance was significantly reduced in pSS compared with controls and DED, whereas normalized IgA2 abundance was reduced in both disease groups compared with controls. Unnormalized ELISA concentrations showed broadly similar patterns, although individual statistical comparisons differed depending on normalization. Two negative correlations involving the IgA1/IgA2 ratio in controls aged ≤50 years remained significant after FDR correction. Analyses stratified by DED subtype, sex, and age, as well as correlation analyses, were exploratory and hypothesis-generating. Given the small and unequal subgroup sizes and potential confounding factors, these findings do not establish subtype-, age-, or sex-specific alterations in IgA subclass profiles.

## 4. Discussion

The present study investigated the distribution of IgA-expressing cells in human ocular tissues and examined IgA subclass profiles in Schirmer strip extracts from controls and patients with DED or pSS. Immunofluorescence analysis demonstrated the presence of CD38^+^IgA^+^ and CD38^−^IgA^+^ cells in the lacrimal gland and eyelid connective tissue beneath the conjunctiva, whereas no IgA-positive cells were detected in the examined corneal sections. IgA1-expressing cells predominated over IgA2-expressing cells in both tissues. Independently, ELISA analysis revealed reduced normalized IgA2 abundance in Schirmer strip extracts from patients with DED and pSS compared with controls, while normalized total IgA abundance was additionally reduced in pSS. These findings provide complementary but independent observations regarding ocular tissue IgA expression and IgA subclass profiles in ocular surface disease. Because tissue samples were obtained from body donors without available clinical characterization and tear samples were collected from separate individuals, no direct relationship between tissue cell populations and disease-associated IgA alterations can be established.

The presence of IgA-expressing cells in the connective tissue surrounding the lacrimal gland acini and beneath the conjunctiva is consistent with the established contribution of ocular-associated tissues to local mucosal immunity. In contrast, no IgA-positive cells were identified in the examined corneal sections. This observation is compatible with the specialized immune environment of the cornea, in which immune responses are tightly regulated to preserve transparency and visual function [29]. Instead, immune surveillance at the ocular surface appears to rely mainly on secretory components of the tear film rather than resident antibody-producing cells within the cornea, similar to mechanisms described in the intestinal mucosa [23].

Within the lacrimal gland and conjunctival region, IgA1-expressing cells were more abundant than IgA2-expressing cells. This distribution resembles the predominance of IgA1 reported in human blood and several mucosal tissues, including the respiratory tract [30,31]. In contrast, IgA2 is relatively enriched at intestinal mucosal sites, where exposure to microbial antigens and bacterial IgA1 proteases may contribute to subclass-specific selection [32,33]. Differences in microbial exposure may contribute to the IgA subclass distribution observed in ocular tissues, with the predominance of IgA1 potentially reflecting the relatively low microbial burden of the ocular surface, as suggested by microbiome studies reporting sparse commensal populations [34]. However, microbial burden was not assessed in the present study, and its contribution to the local balance between IgA1 and IgA2 remains to be established.

In the clinical cohort, total protein concentrations in Schirmer strip extracts were lower in patients with pSS than in patients with DED. This finding is compatible with impaired lacrimal gland secretory function in pSS, which may result from autoimmune-mediated inflammation and tissue damage underlying the pathophysiology of pSS [35,36,37]. In contrast, DED represents a heterogeneous disease in which tear film instability, increased evaporation, and inflammatory processes contribute to ocular surface damage but do not necessarily cause the same degree of lacrimal gland destruction [38,39]. The markedly reduced protein concentrations observed in pSS may therefore reflect more pronounced impairment of lacrimal gland secretory function, potentially associated with autoimmune-mediated tissue damage, including epithelial injury and lymphocytic infiltration of the lacrimal glands. However, protein concentrations measured in Schirmer strip extracts are influenced by several factors beyond glandular secretion, including tear volume, sample collection, extraction efficiency, and the composition of recovered proteins. Consequently, the reduced protein concentrations observed in pSS cannot be attributed exclusively to impaired lacrimal gland function.

Normalization of IgA concentrations to total protein was used to account for differences in recovered protein amounts. Nevertheless, total protein itself differed between disease groups, and normalization to a disease-associated denominator may alter the magnitude or direction of observed differences. To address this concern, unnormalized ELISA concentrations in Schirmer strip extracts were additionally analyzed (Supplementary Figure S8). These analyses showed broadly similar patterns, including reduced IgA2 concentrations in DED and pSS compared with controls and reduced total IgA, IgA1, and IgA2 concentrations in pSS compared with DED. However, individual statistical comparisons differed between normalized and unnormalized analyses. Importantly, the differences in normalized IgA2 abundance between controls and both patient groups remained statistically significant when previously excluded outliers were included in a sensitivity analysis (Supplementary Table S3), supporting the robustness of these statistical findings. Thus, the results support alterations in recovered IgA subclass profiles but do not establish that IgA2 is selectively affected independently of broader changes in tear protein composition.

Exploratory analyses stratified by age and sex revealed additional differences in normalized IgA abundance and subclass ratios. In particular, an increased IgA1/IgA2 ratio was observed in selected older patient groups compared with controls. Such an increase does not necessarily indicate greater IgA1 production or abundance but may instead reflect a proportionally larger reduction in IgA2. Moreover, ratios may be unstable when denominator values are low. Given the small and unequal subgroup sizes, particularly in the pSS cohort (*n *= 10), these observations should be considered exploratory and hypothesis-generating rather than evidence of age- or sex-specific alterations in IgA subclass profiles. Furthermore, the absence of pSS patients aged ≤50 years precludes conclusions regarding age-related differences within this disease group.

Exploratory correlation analyses identified several nominal associations between IgA parameters and clinical measures. After correction for multiple testing using the Benjamini–Hochberg false discovery rate procedure, only two negative correlations remained statistically significant: the IgA1/IgA2 ratio with the Ocular Surface Disease Index and with Schirmer test values, both among controls aged ≤50 years. These findings require cautious interpretation because of the small subgroup sizes and the potential variability of ratio-based measures. They do not establish a causal relationship between IgA subclass composition and ocular surface symptoms or tear secretion.

Several biological processes could potentially contribute to altered IgA profiles at the ocular surface, although none were directly investigated in the present study. Changes in the abundance, differentiation, survival, or secretory activity of IgA-expressing cells may influence local antibody availability. Chronic inflammation and lacrimal gland tissue damage could additionally affect the microenvironment supporting these cells. However, because ocular tissue samples were obtained from body donors and tear samples from independent clinical cohorts, the present findings cannot establish whether disease-associated differences in tear IgA are related to alterations in local IgA-expressing cell populations.

Another potential mechanism involves epithelial IgA transport. Polymeric IgA is transported across epithelial barriers by the polymeric immunoglobulin receptor (pIgR) and released into the tear film as secretory IgA. pIgR expression can be regulated by inflammatory cytokines, including IFN-γ, TNF-α, and IL-4 [40,41,42], which are altered in the tear film and ocular surface tissues of patients with DED and pSS [43,44,45]. Alterations in pIgR expression or function could therefore influence secretory IgA availability. Furthermore, changes in epithelial barrier integrity, vascular permeability, or serum protein leakage may alter the relative contributions of locally produced secretory IgA and serum-derived IgA to the tear film. Since the ELISAs used in this study did not distinguish between these IgA forms, their respective contributions remain unknown.

**Figure 6:**
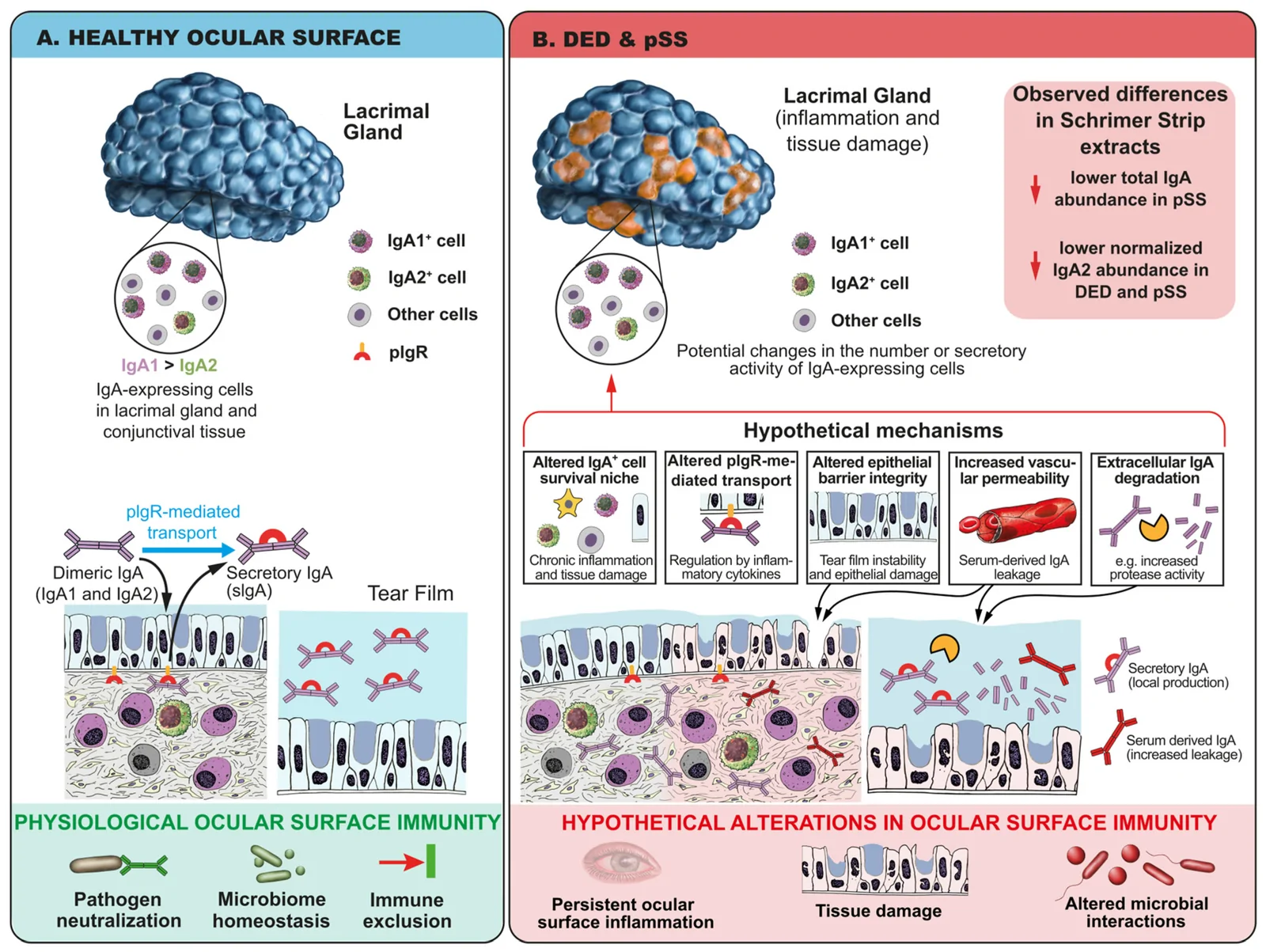
Hypothetical model of IgA homeostasis at the healthy ocular surface and potential alterations in dry eye disease (DED) and primary Sjögren’s syndrome (pSS). (**A**) Under physiological conditions, IgA-expressing cells are present in the lacrimal gland and subconjunctival tissue, with IgA1-expressing cells predominating over IgA2-expressing cells. Polymeric IgA can be transported across epithelial cells via the polymeric immunoglobulin receptor (pIgR) and released into the tear film as secretory IgA, contributing to mucosal immune protection. (**B**) In DED and pSS, several hypothetical mechanisms may contribute to altered ocular surface IgA profiles, including changes in the abundance or secretory activity of IgA-expressing cells, disruption of local cell survival niches, altered pIgR-mediated transport, and increased extracellular IgA degradation. Changes in epithelial barrier integrity and vascular permeability may additionally influence serum-derived IgA leakage and the relative contributions of secretory and non-secretory IgA to the tear film. Interactions between IgA and FcαRI-expressing immune cells may further modulate local inflammatory responses. These mechanisms are hypothesized and were not directly investigated in the present study.

Additional hypothetical mechanisms include altered IgA stability and interactions with local immune cells. IgA may interact with FcαRI-expressing immune cells and modulate inflammatory responses [42,46], although the present findings do not establish a specific pro-inflammatory role for IgA2. Increased tear cathepsin S activity has been reported in Sjögren’s syndrome [47], but direct degradation of ocular surface IgA by this enzyme has not been demonstrated. Accordingly, increased proteolytic degradation remains a possible but unverified explanation for altered IgA abundance. These potential mechanisms, including changes in IgA-expressing cell populations, epithelial transport, barrier integrity, vascular permeability, serum-derived IgA contributions, and extracellular IgA stability, are summarized in the hypothetical model presented in Figure 6. The model illustrates alternative pathways that may influence ocular surface IgA homeostasis rather than mechanisms demonstrated by the present study.

Several limitations should be acknowledged. First, immunofluorescence and tear analyses were performed using independent cohorts, and the clinical status of the tissue donors was unknown, preventing direct associations between tissue IgA-expressing cells and tear IgA profiles. Immunofluorescence analysis was restricted to one section per donor and five randomly selected microscopic fields, potentially limiting the representation of spatial heterogeneity.

Second, different Schirmer strip manufacturers were used for controls and patients, potentially affecting protein recovery and confounding between-group comparisons, particularly because extraction efficiency was not directly compared. Reflex tearing, variable tear volumes, and collection-related dilution may also have influenced the measurements. Since original tear volumes were unavailable, unnormalized values represent concentrations in strip extracts rather than native tear fluid. Moreover, differences in total protein concentrations between groups complicate the interpretation of normalized IgA values. Although all samples were measured within the ELISA standard curve ranges, independent validation of assay accuracy, precision, subclass specificity, and extraction recovery was limited. The assays also did not distinguish secretory from serum-derived IgA.

Finally, differences in age, sex distribution, and medication use may have influenced the observed IgA profiles. Most patients received ocular lubricants, while topical cyclosporine was more frequently documented in pSS and topical corticosteroids and antibiotics predominantly in DED (Supplementary Table S2). Systemic immunomodulatory treatment was also reported in an individual pSS patient. As treatment effects were not systematically evaluated, medication-related confounding cannot be excluded. Small and unequal subgroup sizes further limit the reliability of exploratory comparisons and robust adjustment for potential confounders. Accordingly, findings from age- and sex-stratified analyses should be interpreted as preliminary observations requiring confirmation in larger, appropriately matched cohorts. Future advances in human lacrimal gland organoid technology, ex vivo tissue culture systems, and single-cell approaches, together with larger, better-matched clinical cohorts, standardized tear collection procedures, and independently validated assays, may help overcome these limitations and provide a more comprehensive understanding of IgA regulation and mucosal immune dysfunction at the ocular surface [48,49,50,51,52].

## 5. Conclusions

This study identified IgA-expressing cells in human lacrimal gland and conjunctival tissues, with IgA1-expressing cells predominating over IgA2-expressing cells. Independently, analysis of Schirmer strip extracts revealed lower normalized IgA2 abundance in patients with DED and pSS compared with healthy controls. These findings provide complementary insights into ocular surface IgA homeostasis but do not establish a direct relationship between tissue IgA-expressing cell populations and the observed differences in IgA recovered from Schirmer strip extracts. Differences in collection materials, protein recovery, and patient characteristics preclude attributing these findings exclusively to the underlying diseases. Further studies using standardized collection procedures and well-matched clinical cohorts are needed to clarify the biological relevance and underlying mechanisms of these observations.

## Figures and Tables

**Table 1:** Tissues removed from body donors.

Characteristics	Lacrimal Gland	Eyelid	Cornea
Number of subjects (*n*)	15	15	10
Sex			
Male: (*n*)	7	7	6
Female: (*n*)	8	8	4
Age: years			
Mean ± SD	74 ± 13	78 ± 11	69 ± 11

**Table 2:** Primary antibodies used for immunofluorescence staining.

Antibody	Host Species	Dilution	Stock (mg/mL)	Order-Number	Company
CD38	Rabbit	1:100	0.25	MA5-38376	Thermo Fisher Scientific, Waltham, MA, USA
IgA	Goat	1:100	1.0	2050-01	Southern Biotech, Birmingham, AL, USA
IgA1	Mouse	1:100	0.5	9130-01	Southern Biotech, Birmingham, AL, USA
IgA2	Mouse	1:100	0.5	9140-01	Southern Biotech, Birmingham, AL, USA

**Table 3:** Secondary antibodies used for immunofluorescence staining.

Conjugated Fluorophore	Host Species	Species Reactivity	Dilution	Stock (mg/mL)	Order-Number	Company
Alexa Fluor 488	Donkey	Goat	1:1000	2	A-11055	Invitrogen, Carlsbad, CA, USA
Alexa Fluor 488	Goat	Mouse	1:1000	2	A-11029	Invitrogen, Carlsbad, CA, USA
Alexa Fluor 555	Donkey	Rabbit	1:1000	2	A-31572	Invitrogen, Carlsbad, CA, USA

**Table 4:** Demographic and clinical characteristics of the study cohorts.

Characteristics	Total	Healthy	DED	ADDE	EDE	MDE	pSS
Subjects (*n*)	103	18	75	6	34	35	10
Female: (*n*)	65	8	48	3	21	24	9
Male: (*n*)	38	10	27	3	13	11	1
Age: (years)	-	46 ± 15	55 ± 18	55 ± 21	50 ± 19	61 ± 17	68 ± 10
OSDI-Score	-	4 ± 4	51 ± 20	53 ± 14	50 ± 21	51 ± 21	57 ± 18
Schirmer 1, right eye, (mm/5 min)	-	29 ± 9	11 ± 10	5 ± 4	19 ± 10	5 ± 3	4 ± 6
BUT, right eye, (s)	-	-	5 ± 4	6 ± 6	5 ± 3	4 ± 4	3 ± 1
NEI corneal staining, right eye	-	-	1 ± 2	1 ± 2	1 ± 3	1 ± 1	4 ± 2
Lissamine green staining, right eye	-	-	1 ± 1	2 ± 2	1 ± 1	1 ± 2	5 ± 4

**Table 5:** Antibodies used for quantification of total IgA, IgA1, and IgA2 by ELISA.

Antibody	Application	Host Species	Conjugate	Dilution	Working Concentration (µg/mL)	Order-Number	Company
IgA, F(ab)2	Capture	Goat	-	1:1000	0.5	2052-01	Southern Biotech, Birmingham, AL, USA
IgA-HRP	Detection	Goat	HRP	1:1000	1	2050-05	Southern Biotech, Birmingham, AL, USA
IgA1-HRP	Detection	Mouse	HRP	1:500	2	9130-05	Southern Biotech, Birmingham, AL, USA
IgA2-HRP	Detection	Mouse	HRP	1:500	2	9140-05	Southern Biotech, Birmingham, AL, USA

## Data Availability

The raw data supporting the conclusions of this article will be made available by the authors, without undue reservation.

## Supplementary Materials

The following supporting information can be downloaded at: https://www.mdpi.com/article/10.3390/cells15211971/s1, Figure S1: Total protein concentrations in Schirmer strip extracts from controls and patients with DED and pSS, stratified by sex. (A) Exploratory sex-stratified analysis comparing total protein concentrations between controls and patients with DED or pSS. Total protein concentrations were significantly higher in female patients with DED than in female patients with pSS. Sample sizes after outlier exclusion: female controls (*n *= 8; 0 outliers excluded), female DED (*n *= 48; 0 outliers excluded), female pSS (*n *= 9; 0 outliers excluded), male controls (*n *= 10; 0 outliers excluded), male DED (*n *= 27; 0 outliers excluded), and male pSS (*n *= 1; 0 outliers excluded). (B) Exploratory sex-stratified subgroup analysis of DED patients showed no statistically significant differences between the investigated groups. Sample sizes after outlier exclusion: female controls (*n *= 8; 0 outliers excluded), female ADDE (*n *= 3; 0 outliers excluded), female EDE (*n *= 21; 0 outliers excluded), female MDE (*n *= 24; 0 outliers excluded), female pSS (*n *= 9; 0 outliers excluded), male controls (*n *= 10; 0 outliers excluded), male ADDE (*n *= 3; 0 outliers excluded), male EDE (*n *= 13; 0 outliers excluded), male MDE (*n *= 11; 0 outliers excluded), and male pSS (*n *= 1; 0 outliers excluded). Each data point represents an individual participant. Given the small and unequal subgroup sizes, particularly in the pSS cohort, these sex-stratified findings should be considered exploratory and hypothesis-generating.; Figure S2: Total protein concentrations in Schirmer strip extracts from controls and patients with DED and pSS, stratified by age. (A) Exploratory age-stratified analysis comparing total protein concentrations between controls and patients with DED or pSS. No statistically significant differences were observed between the groups. Sample sizes after outlier exclusion: controls ≤ 50 years (*n *= 9; 0 outliers excluded), controls > 50 years (*n *= 9; 0 outliers excluded), DED ≤ 50 years (*n *= 27; 0 outliers excluded), DED > 50 years (*n *= 48; 0 outliers excluded), pSS ≤ 50 years (*n *= 0; 0 outliers excluded), and pSS > 50 years (*n *= 10; 0 outliers excluded). (B) Exploratory age-stratified subgroup analysis of DED patients showed no statistically significant differences between the groups. Sample sizes after outlier exclusion: controls ≤ 50 years (*n *= 9; 0 outliers excluded), controls > 50 years (*n *= 9; 0 outliers excluded), ADDE ≤ 50 years (*n *= 3; 0 outliers excluded), ADDE >50 years (*n *= 3; 0 outliers excluded), EDE ≤ 50 years (*n *= 16; 0 outliers excluded), EDE > 50 years (*n *= 18; 0 outliers excluded), MDE ≤ 50 years (*n *= 8; 0 outliers excluded), MDE >50 years (*n *= 27; 0 outliers excluded), pSS ≤ 50 years (*n *= 0; 0 outliers excluded), and pSS > 50 years (*n *= 10; 0 outliers excluded). Each data point represents an individual participant. Age-stratified analyses were exploratory. As no patients with pSS were aged ≤50 years, comparisons between age groups within the pSS cohort were not possible.; Figure S3: Correlation analyses of normalized IgA parameters in Schirmer strip extracts and OSDI scores, stratified by age. Total IgA, IgA1, and IgA2 measurements were normalized to total protein content. (A1–J1) No nominally significant correlations were observed between normalized total IgA abundance and OSDI scores. (A2–J2) Normalized IgA1 abundance showed a nominally significant negative correlation with OSDI scores in controls aged ≤50 years and a nominally significant positive correlation in controls aged >50 years. (A3–J3) No nominally significant correlations were observed between normalized IgA2 abundance and OSDI scores. (A4–J4) The IgA1/IgA2 ratio showed a significant negative correlation with OSDI scores in controls aged ≤50 years, which remained statistically significant after FDR correction. Correlations were assessed using Spearman’s rank correlation analysis and were considered exploratory. Except for the negative correlation between the IgA1/IgA2 ratio and OSDI scores in controls aged ≤50 years, none of the correlations remained statistically significant after correction for multiple testing using the Benjamini–Hochberg procedure (FDR = 5%). Unadjusted and FDR-adjusted *p*-values are provided in Supplementary Table S1. Sample sizes: controls ≤ 50 years (*n *= 9), controls > 50 years (*n *= 9), DED ≤ 50 years (*n *= 27), DED >50 years (*n *= 48), pSS ≤ 50 years (*n *= 0), pSS > 50 years (*n *= 10), ADDE ≤ 50 years (*n *= 3), ADDE > 50 years (*n *= 3), EDE ≤ 50 years (*n *= 16), EDE > 50 years (*n *= 18), MDE ≤ 50 years (*n *= 8), and MDE > 50 years (*n *= 27); no outliers were excluded. Each data point represents one individual participant.; Figure S4: Correlation analyses of normalized IgA parameters in Schirmer strip extracts and Schirmer test values, stratified by age. Total IgA, IgA1, and IgA2 measurements were normalized to total protein content. (A1–J1) Normalized total IgA abundance showed a nominally significant negative correlation with Schirmer test values in controls aged ≤50 years. (A2–J2) Nominally significant negative correlations between normalized IgA1 abundance and Schirmer test values were observed in controls and patients with EDE aged ≤50 years. (A3–J3) No nominally significant correlations were observed between normalized IgA2 abundance and Schirmer test values. (A4–J4) The IgA1/IgA2 ratio showed a significant negative correlation with Schirmer test values in controls aged ≤50 years, which remained statistically significant after FDR correction. Correlations were assessed using Spearman’s rank correlation analysis and were considered exploratory. Except for the negative correlation between the IgA1/IgA2 ratio and Schirmer test values in controls aged ≤50 years, none of the correlations remained statistically significant after correction for multiple testing using the Benjamini–Hochberg procedure (FDR = 5%). Unadjusted and FDR-adjusted *p*-values are provided in Supplementary Table S1. Sample sizes: controls ≤ 50 years (*n *= 9), controls > 50 years (*n *= 9), DED ≤ 50 years (*n *= 27), DED > 50 years (*n *= 48), pSS ≤ 50 years (*n *= 0), pSS > 50 years (*n *= 10), ADDE ≤ 50 years (*n *= 3), ADDE > 50 years (*n *= 3), EDE ≤ 50 years (*n *= 16), EDE >50 years (*n *= 18), MDE ≤ 50 years (*n *= 8), and MDE > 50 years (*n *= 27); no outliers were excluded. Each data point represents one individual participant.; Figure S5: Correlation analyses of normalized IgA parameters in Schirmer strip extracts and tear film break-up time (BUT), stratified by age. Total IgA, IgA1, and IgA2 measurements were normalized to total protein content. (A1–J1, A2–J2, A4–J4) No nominally significant correlations were observed between BUT and normalized total IgA abundance, normalized IgA1 abundance, or the IgA1/IgA2 ratio. (A3–J3) Normalized IgA2 abundance showed a nominally significant positive correlation with BUT in patients with pSS aged >50 years and a nominally significant negative correlation in patients with MDE aged >50 years. Correlations were assessed using Spearman’s rank correlation analysis and were considered exploratory. None of the correlations remained statistically significant after correction for multiple testing using the Benjamini–Hochberg procedure (FDR = 5%). Unadjusted and FDR-adjusted *p*-values are provided in Supplementary Table S1. Sample sizes: controls ≤ 50 years (*n *= 9), controls > 50 years (*n *= 9), DED ≤ 50 years (*n *= 27), DED > 50 years (*n *= 48), pSS ≤ 50 years (*n *= 0), pSS > 50 years (*n *= 10), ADDE ≤ 50 years (*n *= 3), ADDE > 50 years (*n *= 3), EDE ≤ 50 years (*n *= 16), EDE > 50 years (*n *= 18), MDE ≤ 50 years (*n *= 8), and MDE > 50 years (*n *= 27); no outliers were excluded. Each data point represents one individual participant.; Figure S6: Correlation analyses of normalized IgA parameters in Schirmer strip extracts and NEI corneal staining scores, stratified by age. Total IgA, IgA1, and IgA2 measurements were normalized to total protein content. (A1–J1, A2–J2, A3–J3) No nominally significant correlations were observed between NEI corneal staining scores and normalized total IgA, IgA1, or IgA2 abundance. (A4–J4) The IgA1/IgA2 ratio showed a nominally significant negative correlation with NEI corneal staining scores in patients with MDE aged ≤50 years. Correlations were assessed using Spearman’s rank correlation analysis and were considered exploratory. None of the correlations remained statistically significant after correction for multiple testing using the Benjamini–Hochberg procedure (FDR = 5%). Unadjusted and FDR-adjusted *p*-values are provided in Supplementary Table S1. Sample sizes: controls ≤ 50 years (*n *= 9), controls > 50 years (*n *= 9), DED ≤ 50 years (*n *= 27), DED > 50 years (*n *= 48), pSS ≤ 50 years (*n *= 0), pSS > 50 years (*n *= 10), ADDE ≤ 50 years (*n *= 3), ADDE > 50 years (*n *= 3), EDE ≤ 50 years (*n *= 16), EDE > 50 years (*n *= 18), MDE ≤ 50 years (*n *= 8), and MDE > 50 years (*n *= 27); no outliers were excluded. Each data point represents one individual participant.; Figure S7: Correlation analyses of normalized IgA parameters in Schirmer strip extracts and Lissamine green staining scores, stratified by age. Total IgA, IgA1, and IgA2 measurements were normalized to total protein content. (A1–J1, A2–J2) Normalized total IgA and IgA1 abundance showed nominally significant negative correlations with Lissamine green staining scores in patients with pSS aged >50 years. (A3–J3, A4–J4) No nominally significant correlations were observed between Lissamine green staining scores and normalized IgA2 abundance or the IgA1/IgA2 ratio. Correlations were assessed using Spearman’s rank correlation analysis and were considered exploratory. None of the correlations remained statistically significant after correction for multiple testing using the Benjamini–Hochberg procedure (FDR = 5%). Unadjusted and FDR-adjusted *p*-values are provided in Supplementary Table S1. Sample sizes: controls ≤ 50 years (*n *= 9), controls > 50 years (*n *= 9), DED ≤ 50 years (*n *= 27), DED > 50 years (*n *= 48), pSS ≤ 50 years (*n *= 0), pSS > 50 years (*n *= 10), ADDE ≤ 50 years (*n *= 3), ADDE > 50 years (*n *= 3), EDE ≤ 50 years (*n *= 16), EDE > 50 years (*n *= 18), MDE ≤ 50 years (*n *= 8), and MDE > 50 years (*n *= 27); no outliers were excluded. Each data point represents one individual participant.; Figure S8: Unnormalized ELISA measurements of total IgA, IgA1 and IgA2 in Schirmer strip extracts. (A–C) Comparison of unnormalized total IgA, IgA1, and IgA2 concentrations in Schirmer strip extracts between controls and patients with DED or pSS. Total IgA and IgA2 concentrations were significantly lower in patients with pSS than in controls, while total IgA, IgA1, and IgA2 concentrations were significantly lower in patients with pSS than in patients with DED. In addition, IgA2 concentrations were significantly lower in patients with DED than in controls. Sample sizes after outlier exclusion: A: controls (*n *= 17; 1 outlier excluded), DED (*n *= 70; 5 outliers excluded), and pSS (*n *= 9; 1 outlier excluded); B: controls (*n *= 18; 0 outliers excluded), DED (*n *= 75; 0 outliers excluded), and pSS (*n *= 10; 0 outliers excluded); C: controls (*n *= 18; 0 outliers excluded), DED (*n *= 66; 9 outliers excluded), and pSS (*n *= 9; 1 outlier excluded). (D–F) Exploratory subgroup analysis comparing unnormalized total IgA, IgA1, and IgA2 concentrations between controls, patients with different DED subtypes, and patients with pSS. Total IgA concentrations were significantly lower in patients with pSS than in controls and patients with EDE or MDE. IgA2 concentrations were also significantly lower in patients with pSS than in controls and patients with EDE. Sample sizes after outlier exclusion: D: controls (*n *= 17; 1 outlier excluded), ADDE (*n *= 6; 0 outliers excluded), EDE (*n *= 30; 4 outliers excluded), MDE (*n *= 34; 1 outlier excluded), and pSS (*n *= 9; 1 outlier excluded); E: controls (*n *= 18; 0 outliers excluded), ADDE (*n *= 6; 0 outliers excluded), EDE (*n *= 34; 0 outliers excluded), MDE (*n *= 35; 0 outliers excluded), and pSS (*n *= 10; 0 outliers excluded); F: controls (*n *= 18; 0 outliers excluded), ADDE (*n *= 5; 1 outlier excluded), EDE (*n *= 30; 4 outliers excluded), MDE (*n *= 33; 2 outliers excluded), and pSS (*n *= 9; 1 outlier excluded). Each data point represents an individual participant; Table S1: Exploratory correlation analyses showing nominally significant associations between normalized IgA parameters and clinical parameters. The table includes correlations with unadjusted *p*-values < 0.05. Spearman’s rank correlation coefficients (r), unadjusted *p*-values, and *p*-values adjusted using the Benjamini–Hochberg procedure are presented. FDR correction was performed separately for each combination of clinical parameter and IgA parameter, resulting in 20 test families. Each test family included all correlations across the investigated study groups and age strata for the respective parameter combination. Two correlations remained statistically significant after FDR correction (5%): the negative correlations between the IgA1/IgA2 ratio and both OSDI scores and Schirmer test values in controls aged ≤50 years. All other nominally significant correlations did not remain significant after FDR correction; Table S2: Topical ocular and systemic immunomodulatory medication use in patients with DED, including ADDE, EDE, and MDE, and pSS. Data are presented as n. Medications were categorized according to therapeutic class. Patients receiving multiple treatments were included in each applicable category. Healthy controls did not use any medication. Systemic medications without immunomodulatory activity and eyelid hygiene products were excluded from this table. Medication use was assessed based on the available clinical records; Table S3: Sensitivity analysis of normalized IgA2 abundance with and without outlier exclusion. Sensitivity analysis of total protein-normalized IgA2 abundance in Schirmer strip extracts from healthy controls, patients with DED, and patients with pSS. The original analysis excluded outliers identified using the ROUT method with Q = 1% (controls: *n *= 3 excluded; DED: *n *= 10 excluded; pSS: *n *= 0 excluded), whereas the sensitivity analysis included all originally measured observations without outlier exclusion. Data distribution was assessed using the Kolmogorov-Smirnov normality test. Group differences were evaluated using the Kruskal-Wallis test followed by Dunn’s multiple comparisons test. Reported *p*-values were adjusted for multiple comparisons using Dunn’s test. Statistical significance was defined as *p* < 0.05.

## Abbreviations

The following abbreviations are used in this manuscript:

DED	Dry eye disease
pSS	Primary Sjögrens’s syndrome
IgA	Immunoglobulin A
EDE	Evaporative dry eye
ADDE	Aqueous-deficient dry eye
MDE	Mixed form of dry eye
pIgR	Polymeric immunoglobulin receptor
PFA	Paraformaldehyde
TBS-T	Tris-buffered saline containing Tween 20
OSDI	Ocular surface disease index
MGD	Meibomian gland dysfunction
BUT	Break-up time
NEI	National Eye Institute corneal fluorescein staining score
LG	Lacrimal gland
Conj.	Conjunctiva
C	Cornea
IFN-γ	Interferon-γ
TNF-α	Tumor necrosis factor-α
IL-4	Interleukin-4
