# Supplementary material for: IgA-Expressing Cells at the Human Ocular Surface and Reduced Tear IgA2 Abundance in Schirmer Strip Extracts from Patients with Dry Eye Disease and Primary Sjögren’s Syndrome
Source: Cells. 2026 Oct 29;15(21):1971. doi: 10.3390/cells15211971 (PMC13762353; doi:10.3390/cells15211971)
Supplement: Supplementary file 1 [file cells-15-01971-s001.zip › cells-4628011-supplementary final.pdf]

Supplementary data

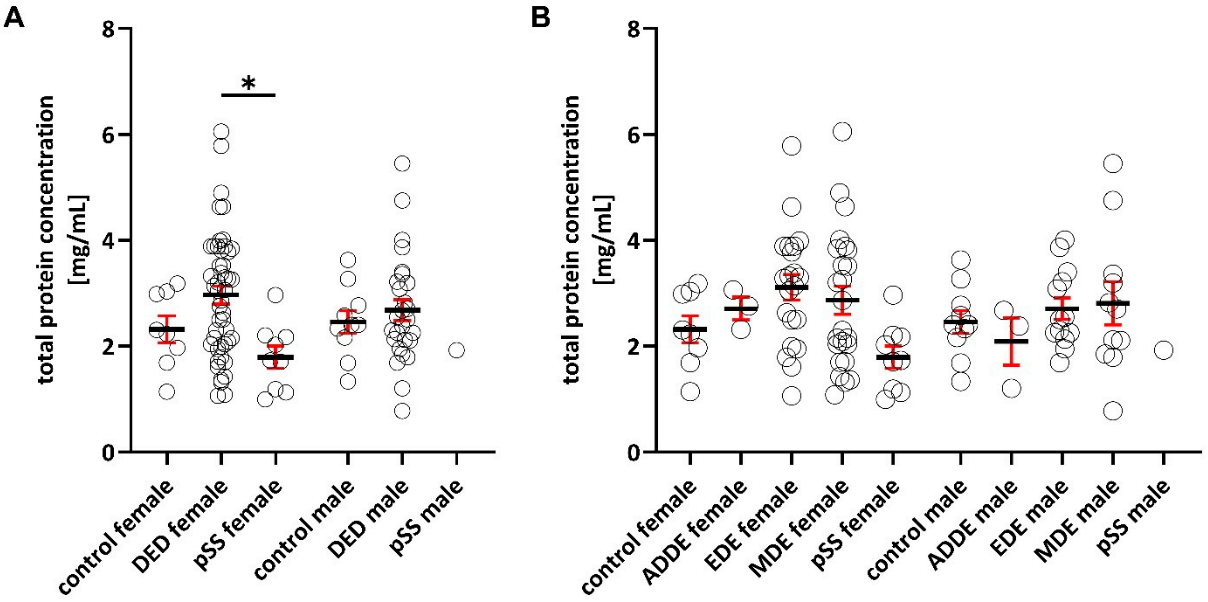

Supplementary Figure S1. Total protein concentrations in Schirmer strip extracts from controls and patients with DED and pSS, stratified by sex. (A) Exploratory sex-stratified analysis comparing total protein concentrations between controls and patients with DED or pSS. Total protein concentrations were significantly higher in female patients with DED than in female patients with pSS. Sample sizes after outlier exclusion: female controls (n = 8; 0 outliers excluded), female DED (n = 48; 0 outliers excluded), female pSS (n = 9; 0 outliers excluded), male controls (n = 10; 0 outliers excluded), male DED (n = 27; 0 outliers excluded), and male pSS (n = 1; 0 outliers excluded). **(B)** Exploratory sex-stratified subgroup analysis of DED patients showed no statistically significant differences between the investigated groups. Sample sizes after outlier exclusion: female controls (n = 8; 0 outliers excluded), female ADDE (n = 3; 0 outliers excluded), female EDE (n = 21; 0 outliers excluded), female MDE (n = 24; 0 outliers excluded), female pSS (n = 9; 0 outliers excluded), male controls (n = 10; 0 outliers excluded), male ADDE (n = 3; 0 outliers excluded), male EDE (n = 13; 0 outliers excluded), male MDE (n = 11; 0 outliers excluded), and male pSS (n = 1; 0 outliers excluded). Each data point represents an individual participant. Given the small and unequal subgroup sizes, particularly in the pSS cohort, these sex-stratified findings should be considered exploratory and hypothesis-generating.

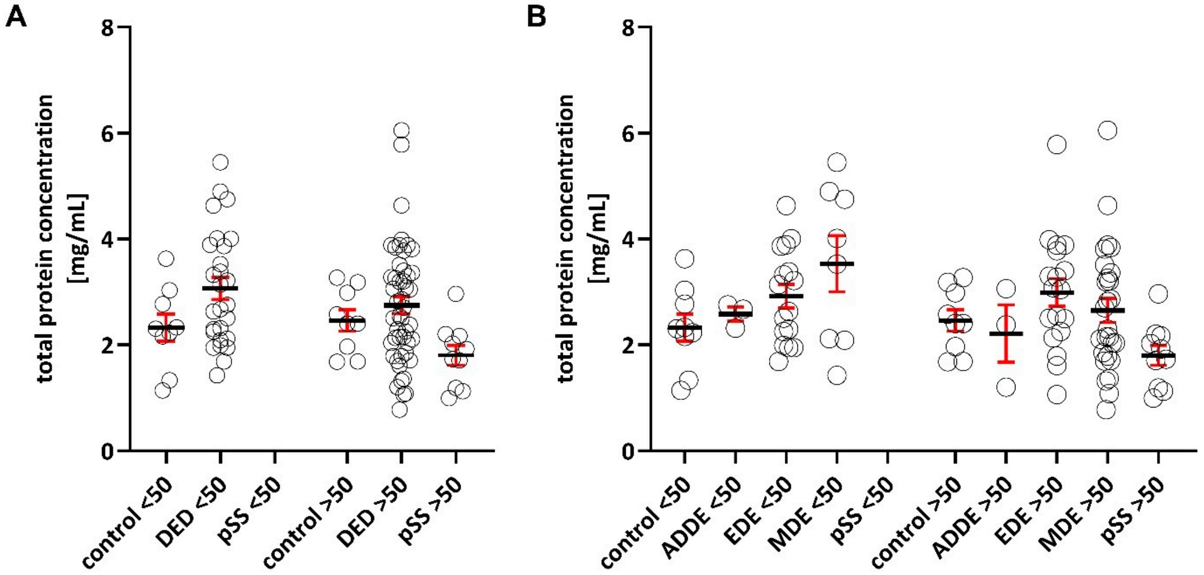

Supplementary Figure S2. Total protein concentrations in Schirmer strip extracts from controls and patients with DED and pSS, stratified by age. (A) Exploratory age-stratified analysis comparing total protein concentrations between controls and patients with DED or pSS. No statistically significant differences were observed between the groups. Sample sizes after outlier exclusion: controls ≤50 years (n = 9; 0 outliers excluded), controls >50 years (n = 9; 0 outliers excluded), DED ≤50 years (n = 27; 0 outliers excluded), DED >50 years (n = 48; 0 outliers excluded), pSS ≤50 years (n = 0; 0 outliers excluded), and pSS >50 years (n = 10; 0 outliers excluded). **(B)** Exploratory age-stratified subgroup analysis of DED patients showed no statistically significant differences between the groups. Sample sizes after outlier exclusion: controls ≤50 years (n = 9; 0 outliers excluded), controls >50 years (n = 9; 0 outliers excluded), ADDE ≤50 years (n = 3; 0 outliers excluded), ADDE >50 years (n = 3; 0 outliers excluded), EDE ≤50 years (n = 16; 0 outliers excluded), EDE >50 years (n = 18; 0 outliers excluded), MDE ≤50 years (n = 8; 0 outliers excluded), MDE >50 years (n = 27; 0 outliers excluded), pSS ≤50 years (n = 0; 0 outliers excluded), and pSS >50 years (n = 10; 0 outliers excluded). Each data point represents an individual participant. Age-stratified analyses were exploratory. As no patients with pSS were aged ≤50 years, comparisons between age groups within the pSS cohort were not possible.

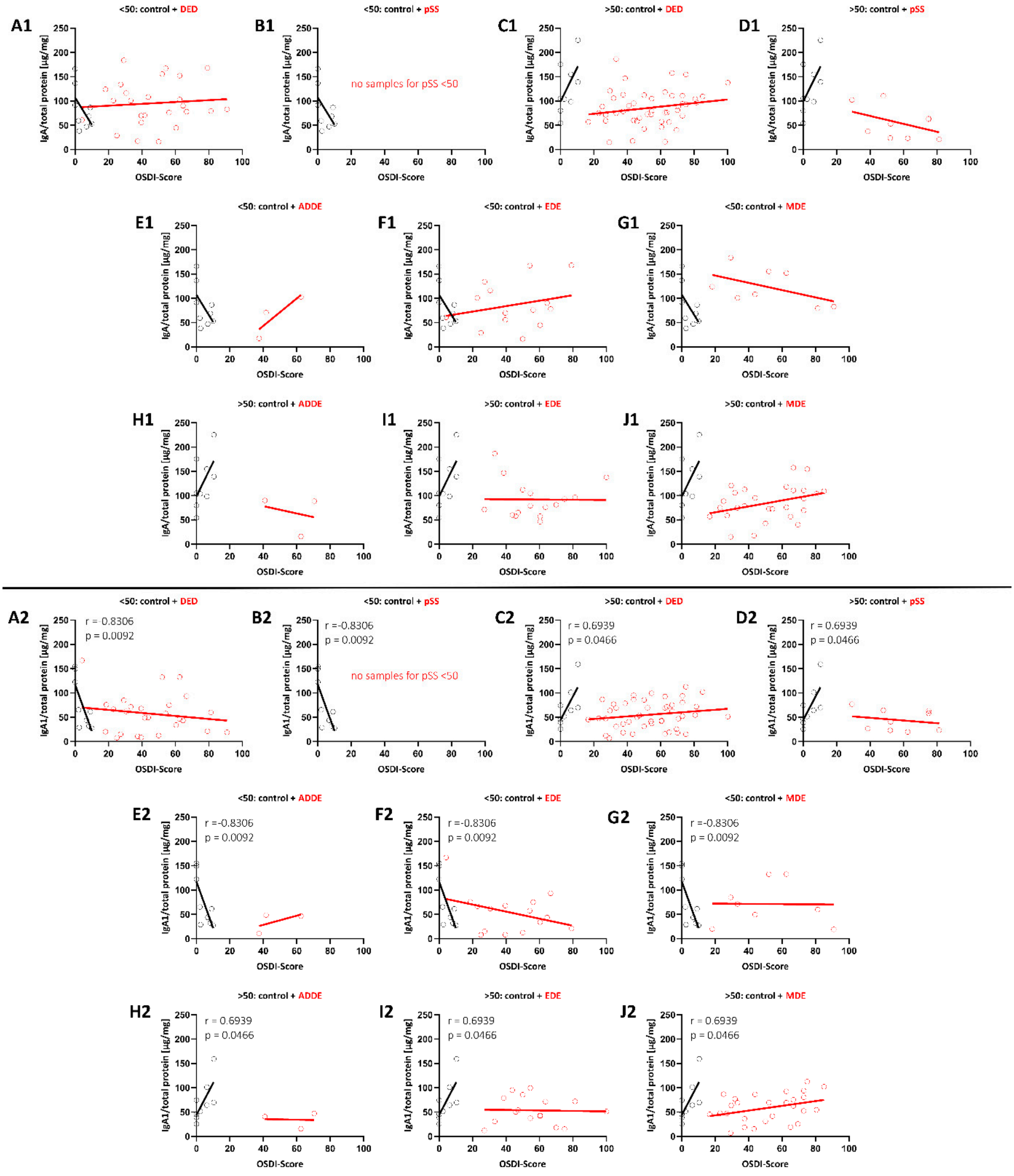
A1
<50: control + DED
B1
<50: control + pSS
no samples for pSS <50
C1
>50: control + DED
D1
>50: control + pSS
E1
<50: control + ADDE
F1
<50: control + EDE
G1
<50: control + MDE
H1
>50: control + ADDE
I1
>50: control + EDE
J1
>50: control + MDE
IgA/total protein [µg/mg]
OSDI-Score
A2
<50: control + DED
r = 0.8306
p = 0.0092
B2
<50: control + pSS
r =-0.8306
p = 0.0092
no samples for pSS <50
C2
>50: control + DED
r = 0.6939
p = 0.0466
D2
>50: control + pSS
r = 0.6939
p = 0.0466
E2
<50: control + ADDE
r =-0.8306
p = 0.0092
F2
<50: control + EDE
r =-0.8306
p = 0.0092
G2
<50: control + MDE
r =-0.8306
p = 0.0092
H2
>50: control + ADDE
r = 0.6939
p = 0.0466
I2
>50: control + EDE
r = 0.6939
p = 0.0466
J2
>50: control + MDE
r = 0.6939
p = 0.0466
IgA1/total protein [µg/mg]
OSDI-Score

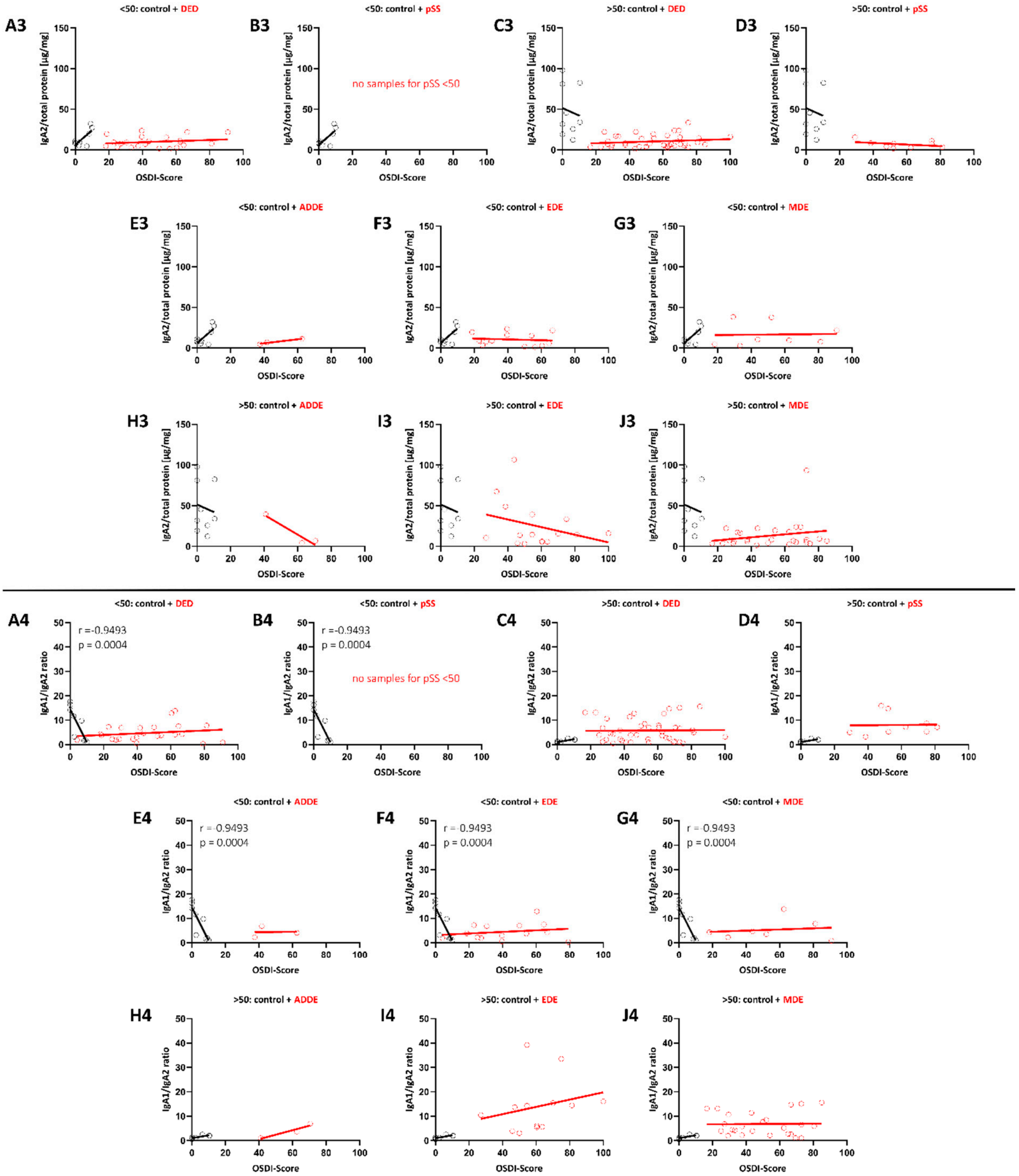

Supplementary Figure S3. Correlation analyses of normalized IgA parameters in Schirmer strip extracts and OSDI scores, stratified by age. Total IgA, IgA1, and IgA2 measurements were normalized to total protein content. **(A1–J1)** No nominally significant correlations were observed between normalized total IgA abundance and OSDI scores. **(A2–J2)** Normalized IgA1 abundance showed a nominally significant negative correlation with OSDI scores in controls aged ≤50 years and a nominally significant positive correlation in controls aged >50 years. **(A3–J3)** No nominally significant correlations were observed between normalized IgA2 abundance and OSDI scores. **(A4–J4)** The IgA1/IgA2 ratio showed a significant negative correlation with

OSDI scores in controls aged ≤50 years, which remained statistically significant after FDR correction. Correlations were assessed using Spearman's rank correlation analysis and were considered exploratory. Except for the negative correlation between the IgA1/IgA2 ratio and OSDI scores in controls aged ≤50 years, none of the correlations remained statistically significant after correction for multiple testing using the Benjamini–Hochberg procedure (FDR = 5%). Unadjusted and FDR-adjusted p-values are provided in Supplementary Table S1. Sample sizes: controls ≤50 years (n = 9), controls >50 years (n = 9), DED ≤50 years (n = 27), DED >50 years (n = 48), pSS ≤50 years (n = 0), pSS >50 years (n = 10), ADDE ≤50 years (n = 3), ADDE >50 years (n = 3), EDE ≤50 years (n = 16), EDE >50 years (n = 18), MDE ≤50 years (n = 8), and MDE >50 years (n = 27); no outliers were excluded. Each data point represents one individual participant.

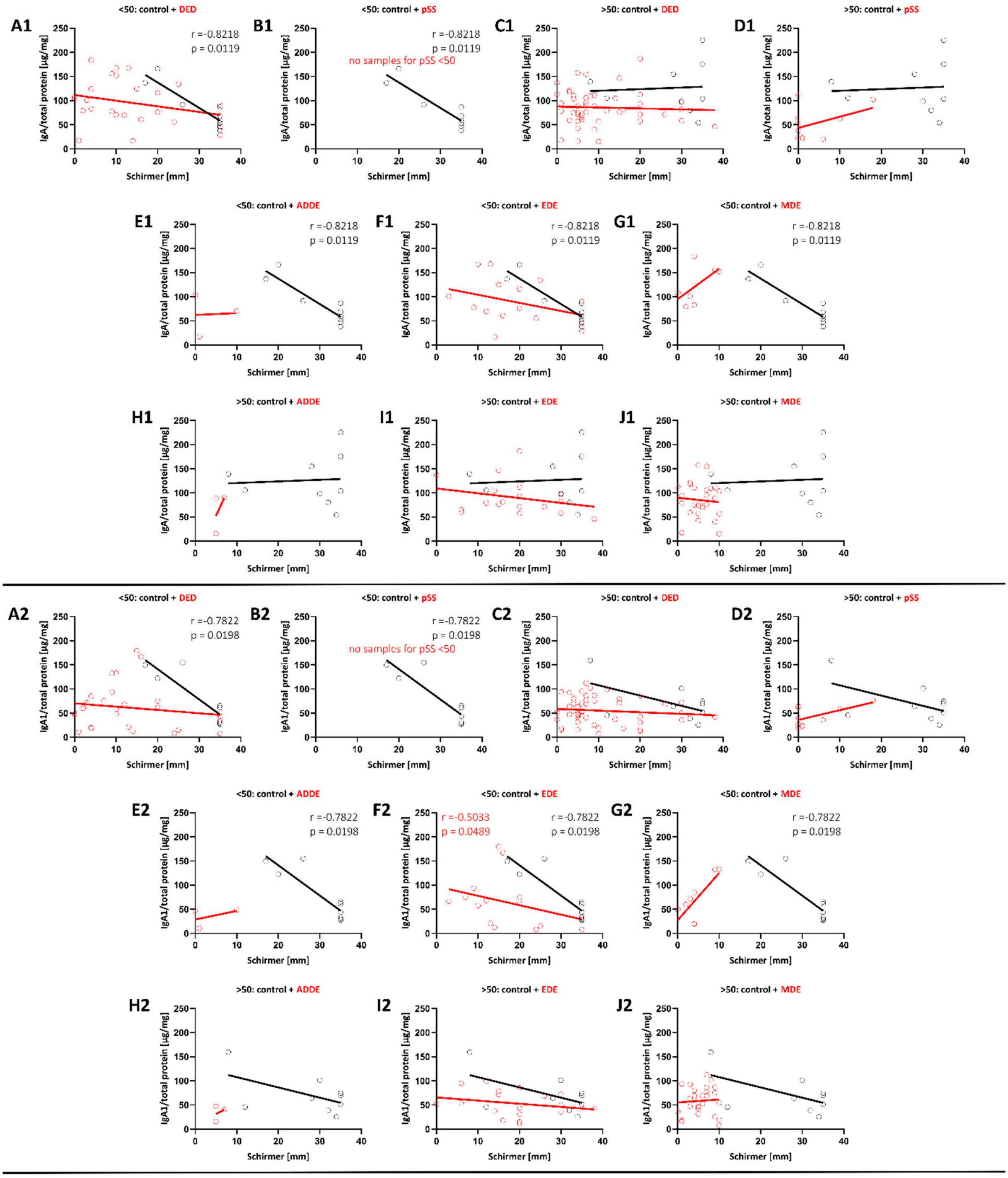
A1
<50: control + DED
r =-0.8218
p = 0.0119
B1
<50: control + pSS
r =-0.8218
p = 0.0119
no samples for pSS <50
C1
>50: control + DED
D1
>50: control + pSS
E1
<50: control + ADDE
r =-0.8218
p = 0.0119
F1
<50: control + EDE
r =-0.8218
p = 0.0119
G1
<50: control + MDE
r =-0.8218
p = 0.0119
H1
>50: control + ADDE
I1
>50: control + EDE
J1
>50: control + MDE
IgA/total protein [µg/mg]
Schirmer [mm]
A2
<50: control + DED
r =-0.7822
p = 0.0198
B2
<50: control + pSS
r =-0.7822
p = 0.0198
no samples for pSS <50
C2
>50: control + DED
D2
>50: control + pSS
E2
<50: control + ADDE
r =-0.7822
p = 0.0198
F2
<50: control + EDE
r =-0.5033
p = 0.0489
r =-0.7822
p = 0.0198
G2
<50: control + MDE
r =-0.7822
p = 0.0198
H2
>50: control + ADDE
I2
>50: control + EDE
J2
>50: control + MDE
IgA1/total protein [µg/mg]
Schirmer [mm]

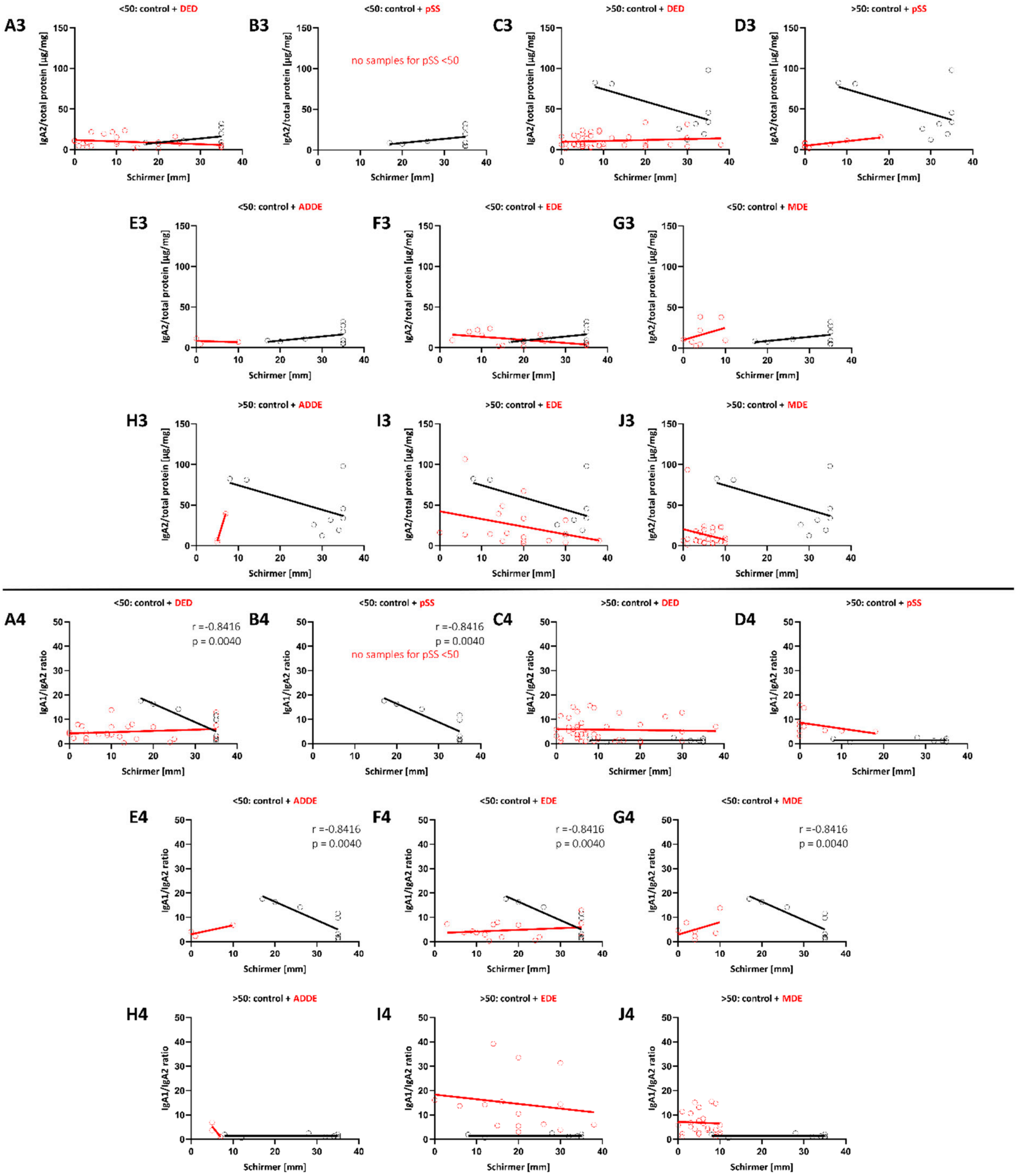

Supplementary Figure S4. Correlation analyses of normalized IgA parameters in Schirmer strip extracts and Schirmer test values, stratified by age. Total IgA, IgA1, and IgA2 measurements were normalized to total protein content. **(A1–J1)** Normalized total IgA abundance showed a nominally significant negative correlation with Schirmer test values in controls aged ≤50 years. **(A2–J2)** Nominally significant negative correlations between normalized IgA1 abundance and Schirmer test values were observed in controls and patients with EDE aged ≤50 years. **(A3–J3)** No nominally significant correlations were observed between normalized IgA2 abundance and Schirmer test values. **(A4–J4)** The IgA1/IgA2 ratio

showed a significant negative correlation with Schirmer test values in controls aged ≤50 years, which remained statistically significant after FDR correction. Correlations were assessed using Spearman's rank correlation analysis and were considered exploratory. Except for the negative correlation between the IgA1/IgA2 ratio and Schirmer test values in controls aged ≤50 years, none of the correlations remained statistically significant after correction for multiple testing using the Benjamini–Hochberg procedure (FDR = 5%). Unadjusted and FDR-adjusted p-values are provided in Supplementary Table S1. Sample sizes: controls ≤50 years (n = 9), controls >50 years (n = 9), DED ≤50 years (n = 27), DED >50 years (n = 48), pSS ≤50 years (n = 0), pSS >50 years (n = 10), ADDE ≤50 years (n = 3), ADDE >50 years (n = 3), EDE ≤50 years (n = 16), EDE >50 years (n = 18), MDE ≤50 years (n = 8), and MDE >50 years (n = 27); no outliers were excluded. Each data point represents one individual participant.

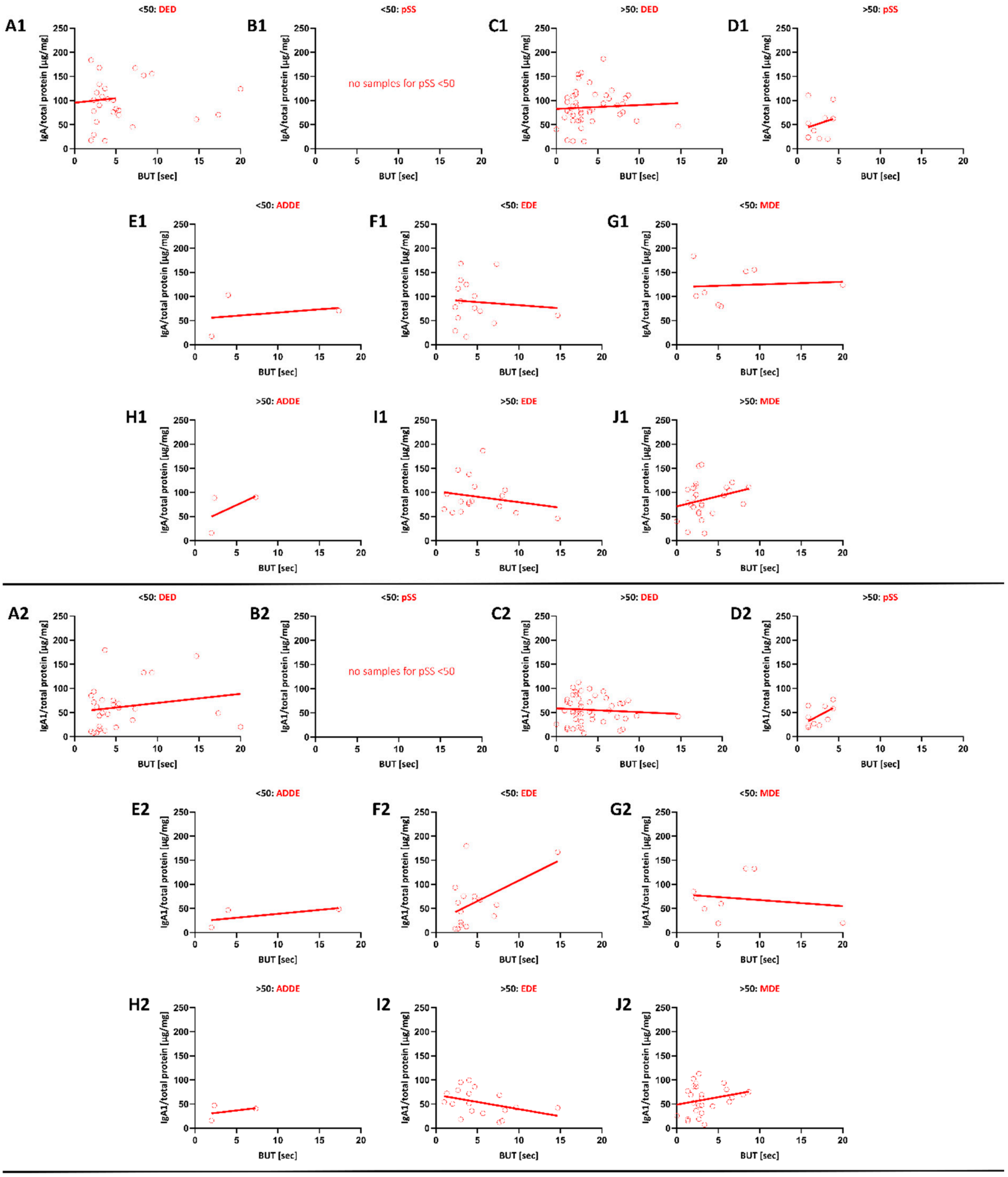
A1
<50: DED
B1
<50: pSS
no samples for pSS <50
C1
>50: DED
D1
>50: pSS
E1
<50: ADDE
F1
<50: EDE
G1
<50: MDE
H1
>50: ADDE
I1
>50: EDE
J1
>50: MDE
IgA/total protein [µg/mg]
BUT [sec]
A2
<50: DED
B2
<50: pSS
no samples for pSS <50
C2
>50: DED
D2
>50: pSS
E2
<50: ADDE
F2
<50: EDE
G2
<50: MDE
H2
>50: ADDE
I2
>50: EDE
J2
>50: MDE
IgA1/total protein [µg/mg]
BUT [sec]

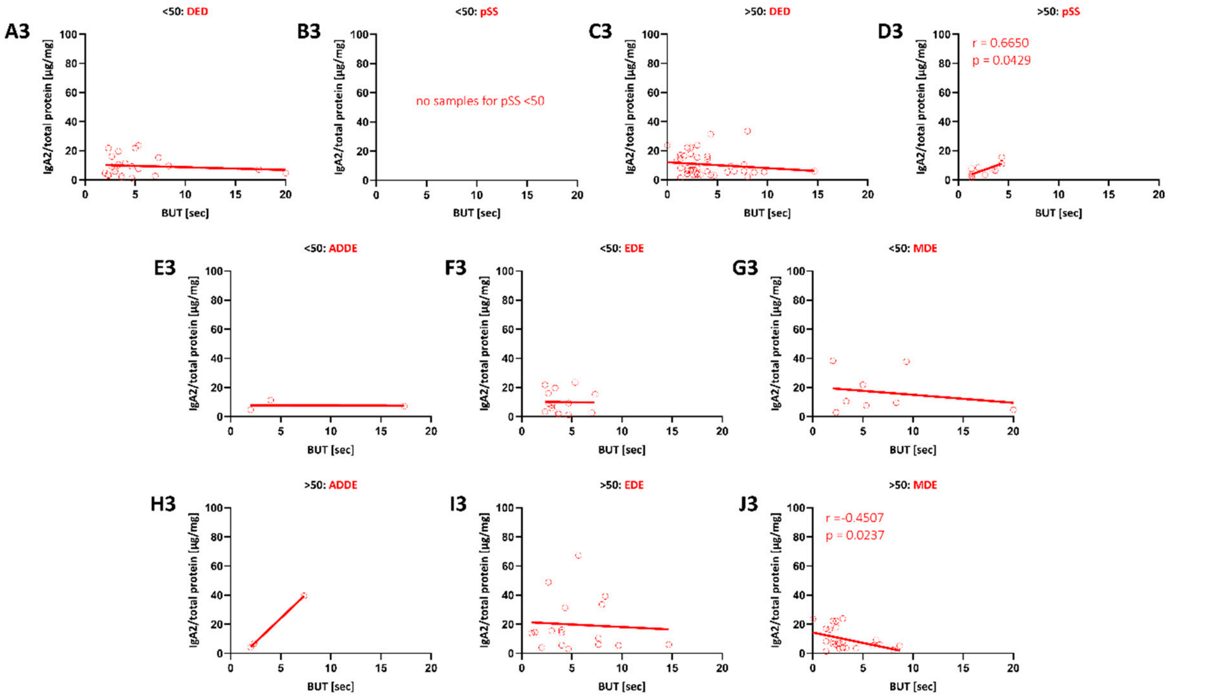

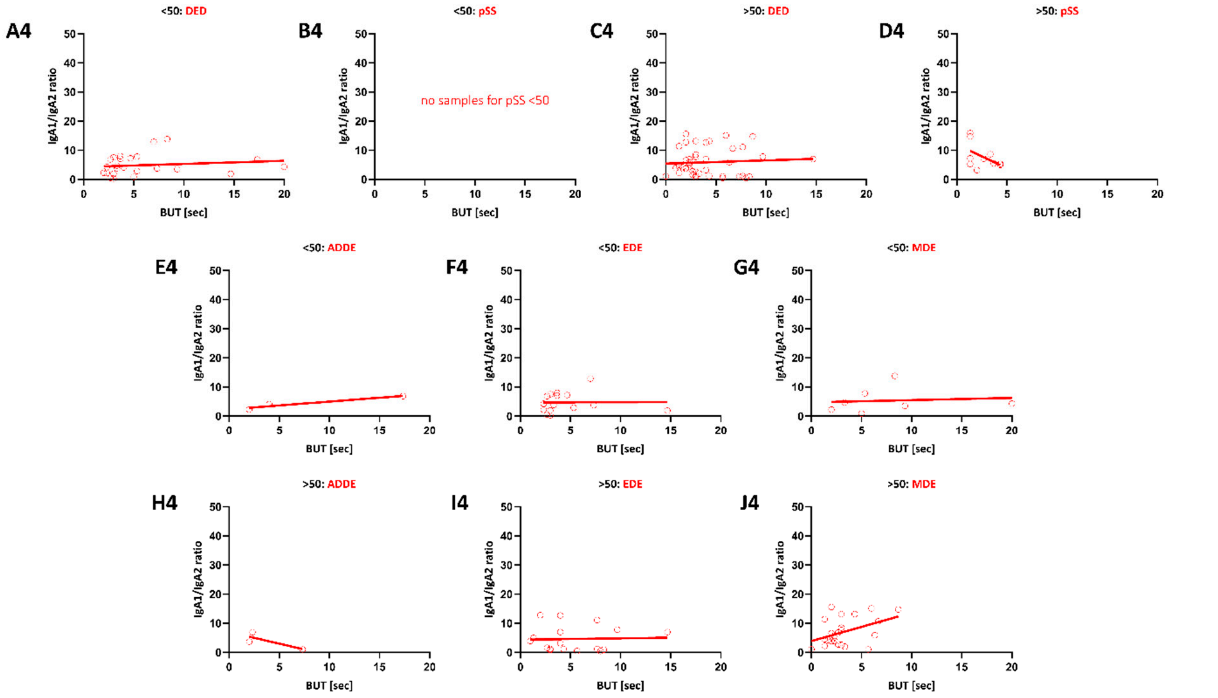

Supplementary Figure S5. Correlation analyses of normalized IgA parameters in Schirmer strip extracts and tear film break-up time (BUT), stratified by age. Total IgA, IgA1, and IgA2 measurements were normalized to total protein content. **(A1–J1, A2–J2, A4–J4)** No nominally significant correlations were observed between BUT and normalized total IgA abundance, normalized IgA1 abundance, or the IgA1/IgA2 ratio. **(A3–J3)** Normalized IgA2 abundance showed a nominally significant positive correlation with BUT in patients with pSS aged >50 years and a nominally significant negative correlation in patients with MDE aged >50 years. Correlations were assessed using Spearman's rank correlation analysis and were considered

exploratory. None of the correlations remained statistically significant after correction for multiple testing using the Benjamini–Hochberg procedure (FDR = 5%). Unadjusted and FDR-adjusted p-values are provided in Supplementary Table S1. Sample sizes: controls ≤50 years (n = 9), controls >50 years (n = 9), DED ≤50 years (n = 27), DED >50 years (n = 48), pSS ≤50 years (n = 0), pSS >50 years (n = 10), ADDE ≤50 years (n = 3), ADDE >50 years (n = 3), EDE ≤50 years (n = 16), EDE >50 years (n = 18), MDE ≤50 years (n = 8), and MDE >50 years (n = 27); no outliers were excluded. Each data point represents one individual participant.

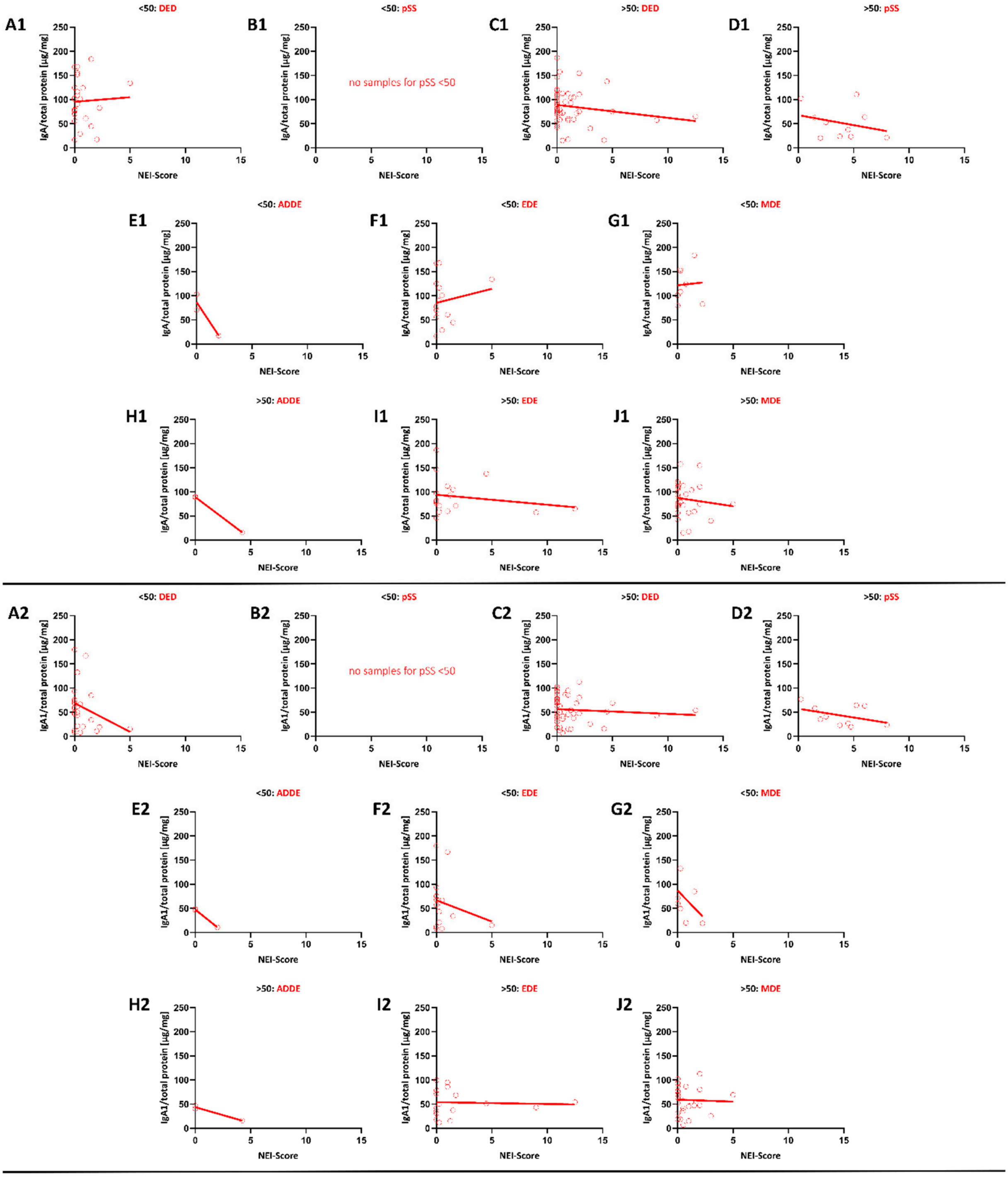

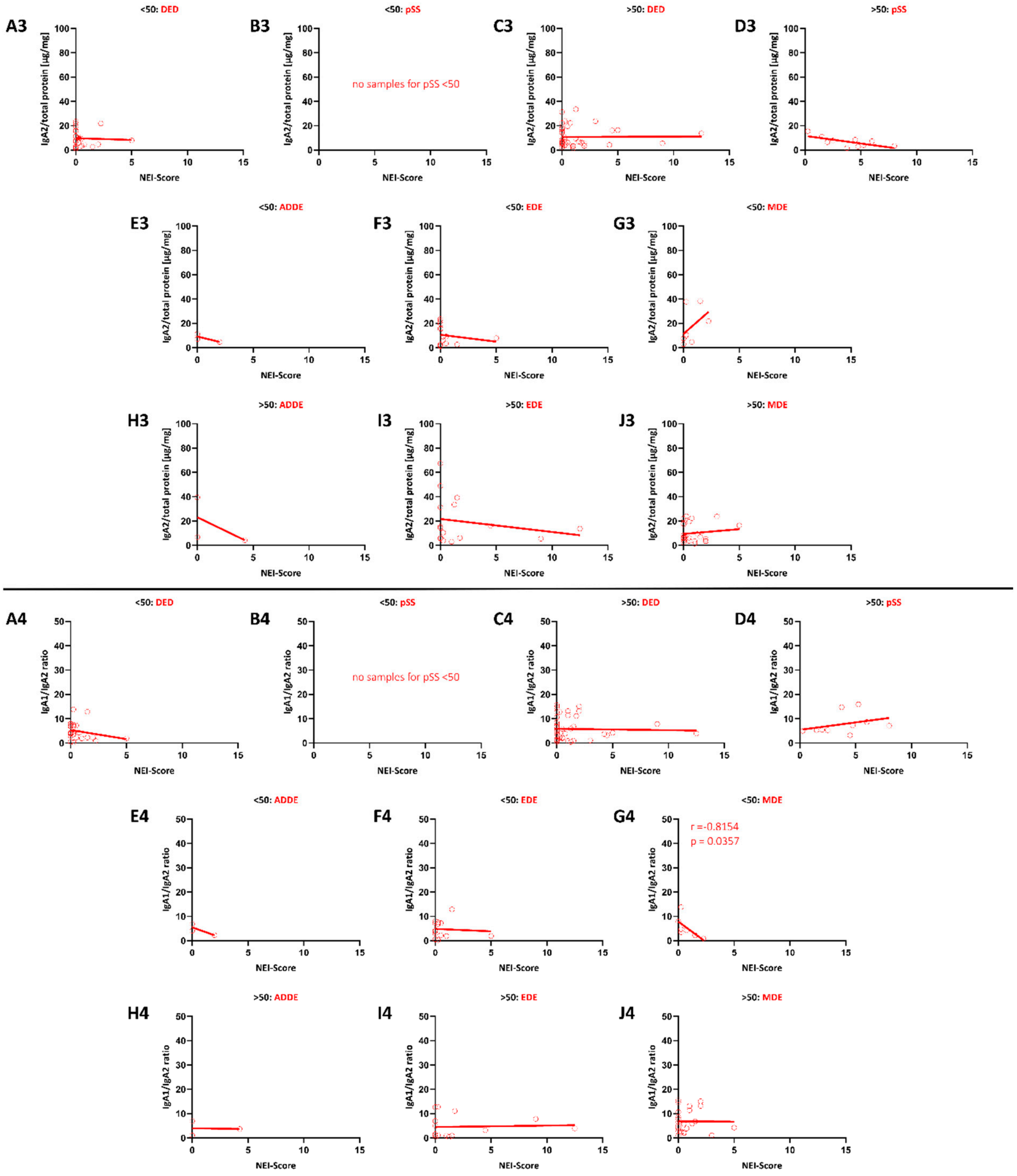

Supplementary Figure S6. Correlation analyses of normalized IgA parameters in Schirmer strip extracts and NEI corneal staining scores, stratified by age. Total IgA, IgA1, and IgA2 measurements were normalized to total protein content. **(A1–J1, A2–J2, A3–J3)** No nominally significant correlations were observed between NEI corneal staining scores and normalized total IgA, IgA1, or IgA2 abundance. **(A4–J4)** The IgA1/IgA2 ratio showed a nominally significant negative correlation with NEI corneal staining scores in patients with MDE aged ≤50 years. Correlations were assessed using Spearman’s rank correlation analysis and were considered exploratory. None of the correlations remained statistically significant after correction for

multiple testing using the Benjamini–Hochberg procedure (FDR = 5%). Unadjusted and FDR-adjusted p-values are provided in Supplementary Table S1. Sample sizes: controls ≤50 years (n = 9), controls >50 years (n = 9), DED ≤50 years (n = 27), DED >50 years (n = 48), pSS ≤50 years (n = 0), pSS >50 years (n = 10), ADDE ≤50 years (n = 3), ADDE >50 years (n = 3), EDE ≤50 years (n = 16), EDE >50 years (n = 18), MDE ≤50 years (n = 8), and MDE >50 years (n = 27); no outliers were excluded. Each data point represents one individual participant.

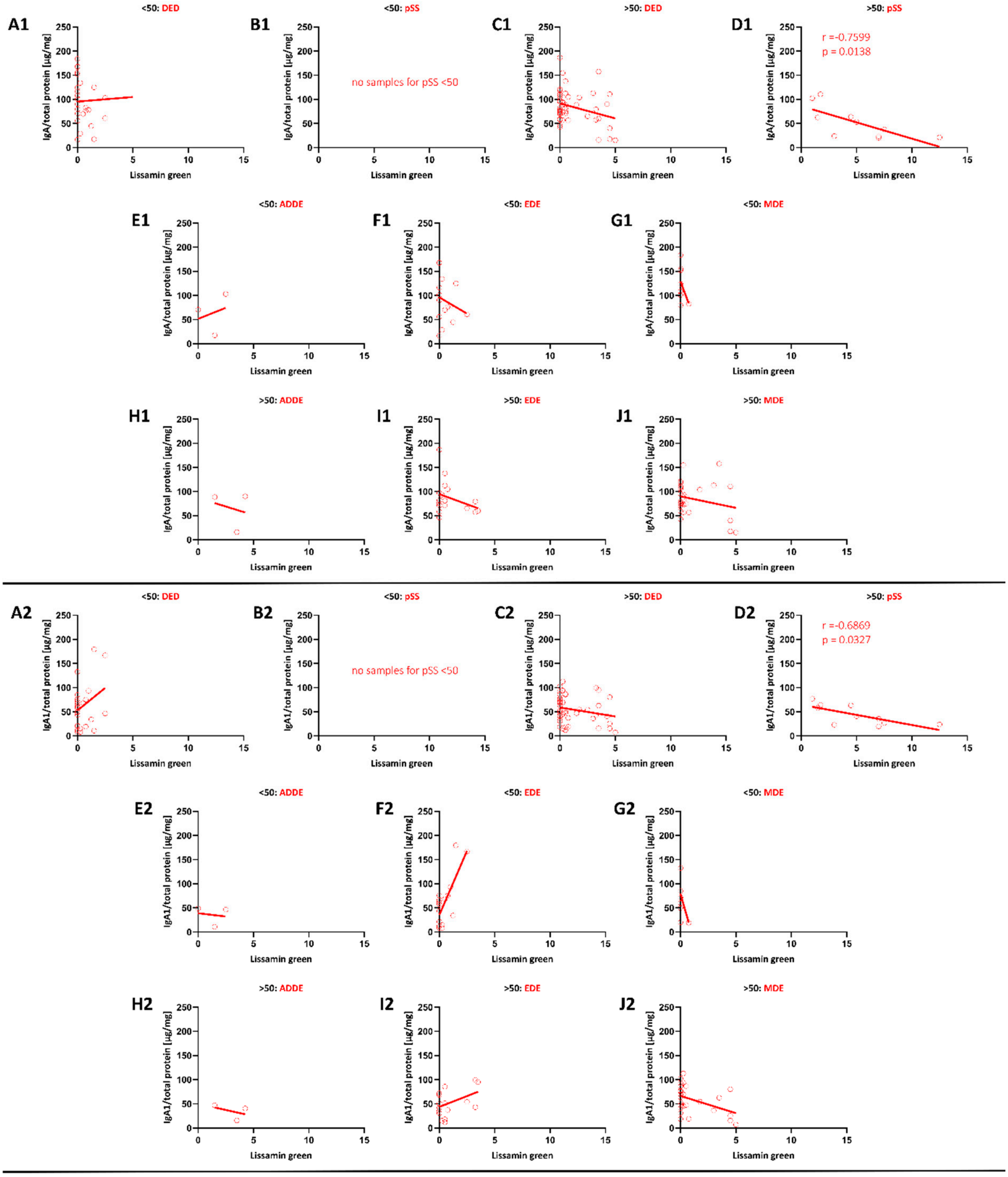

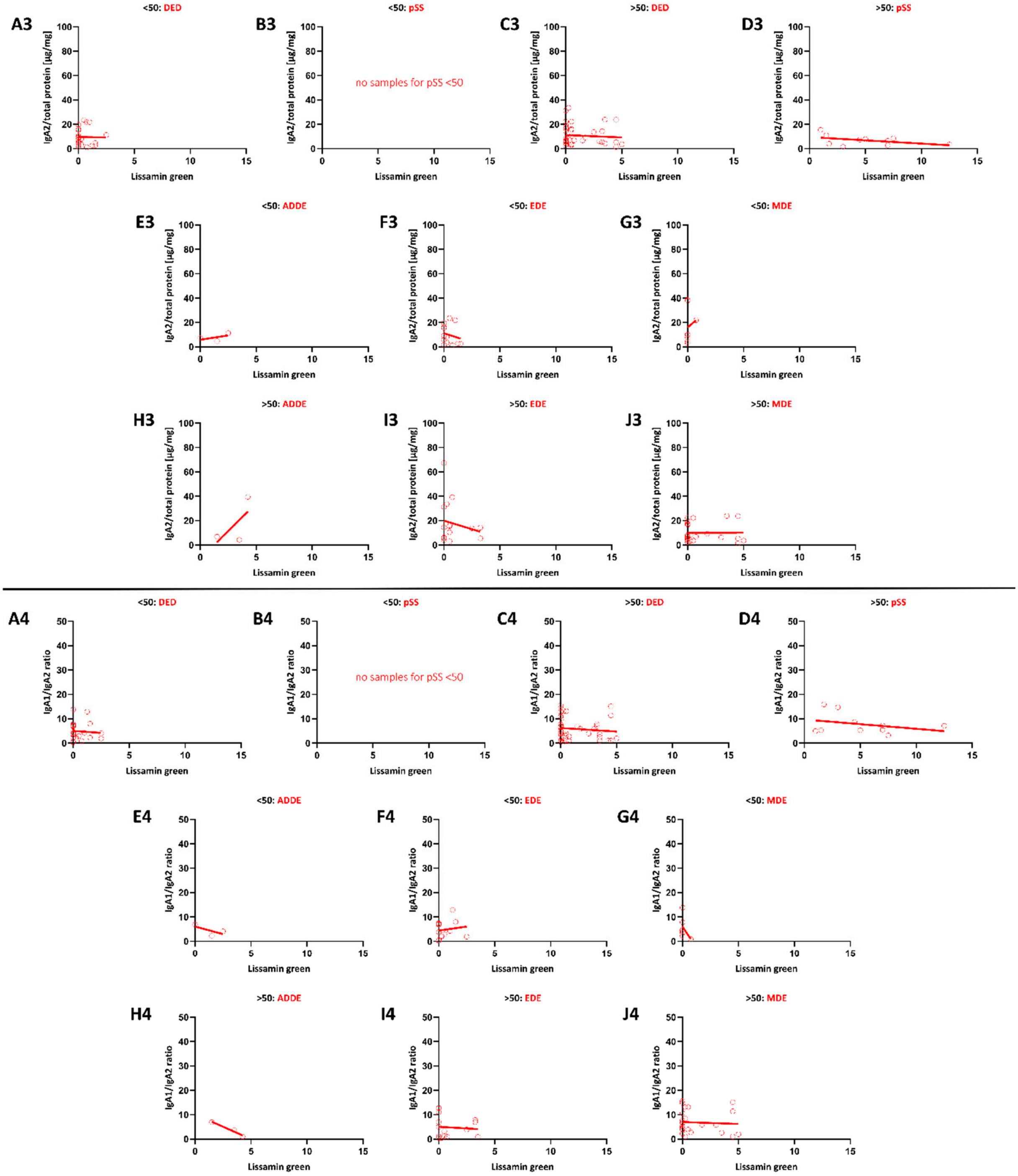

Supplementary Figure S7. Correlation analyses of normalized IgA parameters in Schirmer strip extracts and Lissamine green staining scores, stratified by age. Total IgA, IgA1, and IgA2 measurements were normalized to total protein content. **(A1–J1, A2–J2)** Normalized total IgA and IgA1 abundance showed nominally significant negative correlations with Lissamine green staining scores in patients with pSS aged >50 years. **(A3–J3, A4–J4)** No nominally significant correlations were observed between Lissamine green staining scores and normalized IgA2 abundance or the IgA1/IgA2 ratio. Correlations were assessed using Spearman's rank correlation analysis and were considered exploratory. None of the correlations remained

statistically significant after correction for multiple testing using the Benjamini–Hochberg procedure (FDR = 5%). Unadjusted and FDR-adjusted p-values are provided in Supplementary Table S1. Sample sizes: controls ≤50 years (n = 9), controls >50 years (n = 9), DED ≤50 years (n = 27), DED >50 years (n = 48), pSS ≤50 years (n = 0), pSS >50 years (n = 10), ADDE ≤50 years (n = 3), ADDE >50 years (n = 3), EDE ≤50 years (n = 16), EDE >50 years (n = 18), MDE ≤50 years (n = 8), and MDE >50 years (n = 27); no outliers were excluded. Each data point represents one individual participant.

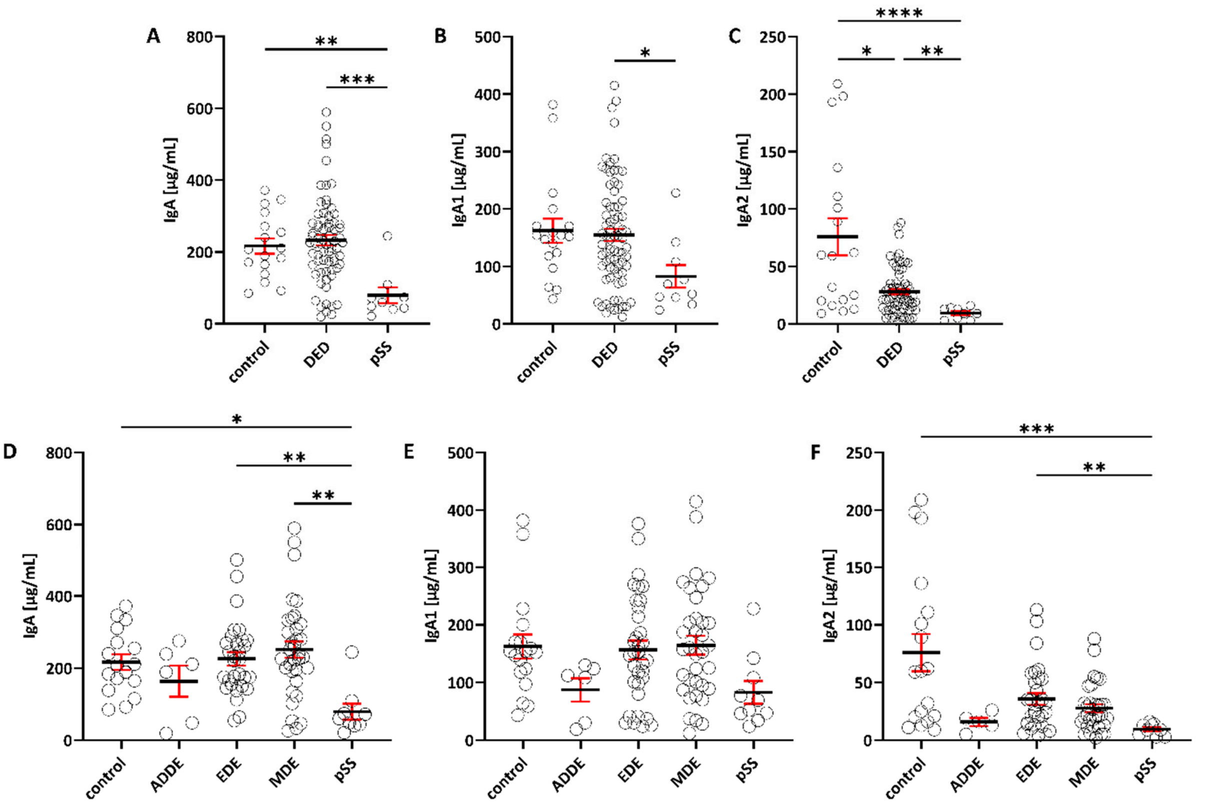

Supplementary Figure S8. Unnormalized ELISA measurements of total IgA, IgA1 and IgA2 in Schirmer strip extracts. (A-C) Comparison of unnormalized total IgA, IgA1, and IgA2 concentrations in Schirmer strip extracts between controls and patients with DED or pSS. Total IgA and IgA2 concentrations were significantly lower in patients with pSS than in controls, while total IgA, IgA1, and IgA2 concentrations were significantly lower in patients with pSS than in patients with DED. In addition, IgA2 concentrations were significantly lower in patients with DED than in controls. Sample sizes after outlier exclusion: A: controls (n = 17; 1 outlier excluded), DED (n = 70; 5 outliers excluded), and pSS (n = 9; 1 outlier excluded); B: controls (n = 18; 0 outliers excluded), DED (n = 75; 0 outliers excluded), and pSS (n = 10; 0 outliers excluded); C: controls (n = 18; 0 outliers excluded), DED (n = 66; 9 outliers excluded), and pSS (n = 9; 1 outlier excluded). **(D-F)** Exploratory subgroup analysis comparing unnormalized total

IgA, IgA1, and IgA2 concentrations between controls, patients with different DED subtypes, and patients with pSS. Total IgA concentrations were significantly lower in patients with pSS than in controls and patients with EDE or MDE. IgA2 concentrations were also significantly lower in patients with pSS than in controls and patients with EDE. Sample sizes after outlier exclusion: D: controls (n = 17; 1 outlier excluded), ADDE (n = 6; 0 outliers excluded), EDE (n = 30; 4 outliers excluded), MDE (n = 34; 1 outlier excluded), and pSS (n = 9; 1 outlier excluded); E: controls (n = 18; 0 outliers excluded), ADDE (n = 6; 0 outliers excluded), EDE (n = 34; 0 outliers excluded), MDE (n = 35; 0 outliers excluded), and pSS (n = 10; 0 outliers excluded); F: controls (n = 18; 0 outliers excluded), ADDE (n = 5; 1 outlier excluded), EDE (n = 30; 4 outliers excluded), MDE (n = 33; 2 outliers excluded), and pSS (n = 9; 1 outlier excluded). Each data point represents an individual participant.

Supplementary Table S1. Exploratory correlation analyses showing nominally significant associations between normalized IgA parameters and clinical parameters. The table includes correlations with unadjusted p-values <0.05. Spearman's rank correlation coefficients (r), unadjusted p-values, and p-values adjusted using the Benjamini–Hochberg procedure are presented. FDR correction was performed separately for each combination of clinical parameter and IgA parameter, resulting in 20 test families. Each test family included all correlations across the investigated study groups and age strata for the respective parameter combination. Two correlations remained statistically significant after FDR correction (5%): the negative correlations between the IgA1/IgA2 ratio and both OSDI scores and Schirmer test values in controls aged ≤50 years. All other nominally significant correlations did not remain significant after FDR correction.

**Clinical parameter**	**Study group**	**IgA parameter**	**Spearman's r**	**Unadjusted p**	**FDR-adjusted p**
OSDI	≤50 control	IgA1	-0,8306	0,0092	0,1012
OSDI	>50 control	IgA1	0,6939	0,0466	0,2482
OSDI	≤50 control	IgA1/IgA2 ratio	-0,9493	0,0004	0,0044
Schirmer value	≤50 control	IgA	-0,8218	0,0119	0,1309
Schirmer value	≤50 control	IgA1	-0,7822	0,0198	0,2178
Schirmer value	≤50 EDE	IgA1	-0,5033	0,0489	0,2690
Schirmer value	≤50 control	IgA1/IgA2 ratio	-0,8416	0,0040	0,0440
BUT	>50 pSS	IgA2	0,6650	0,0429	0,1931
BUT	>50 MDE	IgA2	-0,4507	0,0237	0,1931

NEI	≤50 MDE	IgA1/IgA2 ratio	-0,8154	0,0357	0,3213
Lissamine green	>50 pSS	IgA	-0,7599	0,0138	0,1242
Lissamine green	>50 pSS	IgA1	-0,6869	0,0327	0,2286

Supplementary Table S2. Topical ocular and systemic immunomodulatory medication use in patients with DED, including ADDE, EDE, and MDE, and pSS. Data are presented as n. Medications were categorized according to therapeutic class. Patients receiving multiple treatments were included in each applicable category. Healthy controls did not use any medication. Systemic medications without immunomodulatory activity and eyelid hygiene products were excluded from this table. Medication use was assessed based on the available clinical records.

**Medication category**	**DED (n=75)**	**ADDE (n=6)**	**EDE (n=34)**	**MDE (n=35)**	**pSS (n=10)**
Artificial tears/ocular lubricants	60	5	27	28	9
Topical cyclosporine	6	1	2	3	6
Topical corticosteroids	11	1	5	5	-
Topical antibiotics/antibiotic-containing preparations	6	-	5	1	-
Antiglaucoma eye drops	2	1	-	1	-
Autologous serum eye drops	-	-	-	-	1
Albumin-containing eye drops	1	-	1	-	2
Systemic immunomodulatory therapy	-	-	-	-	1
No topical medication documented	13	1	6	6	-

Supplementary Table S3. Sensitivity analysis of normalized IgA2 abundance with and without outlier exclusion. Sensitivity analysis of total protein-normalized IgA2 abundance in Schirmer strip extracts from healthy controls, patients with DED, and patients with pSS. The original analysis excluded outliers identified using the ROUT method with Q = 1% (controls: n = 3 excluded; DED: n = 10 excluded; pSS: n = 0 excluded), whereas the sensitivity analysis included all originally measured observations without outlier exclusion. Data distribution was assessed using the Kolmogorov-Smirnov normality test. Group differences were evaluated using the Kruskal-Wallis test followed by Dunn's multiple comparisons test. Reported p-values were adjusted for multiple comparisons using Dunn's test. Statistical significance was defined as $p < 0.05$.

Group comparison	Excluding outliers (p-value)	Including all observations (p-value)
Controls vs. DED	0.0106	0.0245
Controls vs. pSS	0.0085	0.0040
DED vs. pSS	0.7755	0.2683
